# Identification and characterization of *Faecalibacterium* prophages rich in diversity-generating retroelements

**DOI:** 10.1128/spectrum.01066-24

**Published:** 2024-12-31

**Authors:** Anastasia Gulyaeva, Lei Liu, Sanzhima Garmaeva, Marloes Kruk, Rinse K. Weersma, Hermie J. M. Harmsen, Alexandra Zhernakova

**Affiliations:** 1 Department of Genetics, University Medical Center Groningen, Groningen, the Netherlands; 2 Department of Medical Microbiology, University Medical Center Groningen, Groningen, the Netherlands; 3 Department of Gastroenterology and Hepatology, University Medical Center Groningen, Groningen, the Netherlands; University of California San Diego, La Jolla, California, USA

**Keywords:** bacteriophage, DGR, microbiome

## Abstract

Metagenomics has revealed the incredible diversity of phages within the human gut. However, very few of these phages have been subjected to in-depth experimental characterization. One promising method of obtaining novel phages for experimental characterization is through induction of the prophages integrated into the genomes of cultured gut bacteria. Here, we developed a bioinformatic approach to prophage identification that builds on prophage genomic properties, existing prophage-detecting software, and publicly available virome sequencing data. We applied our approach to 22 strains of bacteria belonging to the genus *Faecalibacterium*, resulting in identification of 15 candidate prophages, and validated the approach by demonstrating the activity of five prophages from four of the strains. The genomes of three active phages were identical or similar to those of known phages, while the other two active phages were not represented in the Viral RefSeq database. Four of the active phages possessed a diversity-generating retroelement (DGR), and one retroelement had two variable regions. DGRs of two phages were active at the time of the induction experiments, as evidenced by nucleotide variation in sequencing reads. We also predicted that the host range of two active phages may include multiple bacterial species. Finally, we noted that four phages were less prevalent in the metagenomes of inflammatory bowel disease patients compared to a general population cohort, a difference mainly explained by differences in the abundance of the host bacteria. Our study highlights the utility of prophage identification and induction for unraveling phage molecular mechanisms and ecological interactions.

**IMPORTANCE:**

While hundreds of thousands of phage genomes have been discovered in metagenomics studies, only a few of these phages have been characterized experimentally. Here, we explore phage characterization through bioinformatic identification of prophages in genomes of cultured bacteria, followed by prophage induction. Using this approach, we detect the activity of five prophages in four strains of commensal gut bacteria *Faecalibacterium*. We further note that four of the prophages possess diversity-generating retroelements implicated in rapid mutation of phage genome loci associated with phage–host and phage–environment interactions and analyze the intricate patterns of retroelement activity. Our study highlights the potential of prophage characterization for elucidating complex molecular mechanisms employed by the phages.

## INTRODUCTION

Phages—the viruses of bacteria—are an integral component of the human gut ecosystem. All phages rely on their bacterial hosts for survival and replication, but differ in terms of phage particle morphology, genome organization, and infectious cycle. For example, phages can be classified as virulent or temperate depending on their ability to lysogenize their host and become a prophage. Phage–bacterium interactions, which can range from antagonistic to mutually beneficial, shape the bacterial community of the gut, playing an indispensable role in maintaining human gut homeostasis (1).

In recent years, metagenomic sequencing has revealed the incredible diversity of human gut phages. The number of gut-associated phage species discovered through metagenomics is estimated to exceed 50,000 and continues to increase (2). At the same time, there is a striking discrepancy between the large number of metagenomically assembled phage genomes and the small number of phages that have been characterized experimentally. For example, in the IMG/VR 4.1 database alone, there are 38,202 genomes representing distinct crAss-like phages (a group highly abundant in the human gut) (3–6), yet only a few dozens of crAss-like phages have been isolated in a culture of host cells (7–11). And while metagenomically assembled genomes contain a treasure trove of information about phage biology, the isolation of phages is essential for in-depth experimental characterization of the molecular mechanisms they employ.

One of the molecular mechanisms that can be further explored through experimental characterization is targeted hypermutation by diversity-generating retroelements (DGRs). DGRs, which are employed by phages and prokaryotes, include a template genome repeat (TR), a variable genome repeat (VR), and a reverse transcriptase (RT) gene (12–15). Moreover, a DGR may include multiple VRs (12, 16–18). The TR transcript serves as a template for adenine-specific error-prone reverse transcription by the RT, and the resulting complementary DNA replaces the VR. Acquired VR mutations then translate into amino acid substitutions in the target protein encoded by the VR-containing gene (12). Target proteins of phages have been suggested to mediate binding to host cells and environmental materials, with DGRs serving to optimize binding specificities (13). For example, the target protein of the *Bordetella* phage BPP-1 is the major tropism determinant (MTD), a structural protein that binds to a host cell receptor prior to host cell entry. The BPP-1 DGR was shown to switch BPP-1 tropism in response to host phase variation (12).

Further research on DGRs and other phage-related phenomena could benefit from the availability of a wide range of phages for experimental characterization (19). One promising method of obtaining novel phages for experimental characterization is through induction of prophages lysogenizing cultured gut bacteria (17, 20–22). This method is cost-effective as existing bacterial culture collections can be utilized. It also allows to preselect bacterial cultures harboring prophages of interest based on bioinformatic analysis. Here, we propose a bioinformatic prophage identification approach that builds on the rich publicly available virus metagenomic data. We then experimentally validate our approach by demonstrating the activity of five prophages from four strains of *Faecalibacterium* (23), a commensal bacterium of the human gut whose abundance is reduced in different intestinal disorders (24). Finally, we use the resulting data to analyze patterns of phage DGR activity.

## MATERIALS AND METHODS

### Bacterial strain sources

Out of the 22 *Faecalibacterium* strains analyzed in this study (Table S1), four strains, namely, A2-165, L2-15, L2-6, and L2-61, were a kind gift of Silvia Duncan of the Rowett Institute of Nutrition and Health (Aberdeen, United Kingdom), the laboratory where they were isolated (25, 26). The stains were subcultured in our laboratory for more than 10 years. The other 18 strains were isolated in our laboratory between 2009 and 2018 (27).

### Bacterial genome sequencing

All isolates were grown on YCFAG agar plates for 24–48 hours. For each isolate, several colonies (about 5 µL) of the culture were suspended in 300 µL microbead solution, which was subjected to DNA extraction with the Ultraclean Microbial DNA isolation kit (Mo Bio Laboratories, Carlsbad, CA, USA). DNA concentration and purity were measured using a NanoDrop 2000c spectrophotometer (Thermo Fisher Scientific, Waltham, MA, USA) and the Qubit double-stranded DNA (dsDNA) HS and BR assay kits (Life Technologies, Carlsbad, CA, USA). One nanogram of bacterial DNA was used for library preparation. The DNA library was prepared using the Nextera XT library preparation kit with the Nextera XT v2 index kit (Illumina, San Diego, CA, USA). The library fragment length was aimed at fragments with a median size of 575 bp and was assessed with the Genomic DNA ScreenTape assay with the 2200 Tape-Station system (Agilent Technologies, Waldbronn, Germany). Subsequently, the library was sequenced on a MiSeq sequencer using a 2 × 250 (500v2) cartridge and the MiSeq reagent kit v2, generating 250-bp paired-end reads (Illumina).

### Prophage induction

The *Faecalibacterium* strains used for prophage induction in this study, A2-165, FM8, HTF-162, L2-61, HTF-238, and HTF-128, were from our local strain collection (Department of Medical Microbiology, University Medical Center Groningen, Groningen, the Netherlands). Growth experiments were carried out anaerobically at 37°C in a Bactron 600 anaerobic incubator (Kentrom Microbiome BV). Culturing in the presence of mitomycin C (MMC) and heat shock were used as induction stimuli. The final MMC concentrations (µg/mL) of 0, 0.1, 2, 6, 8, 12, and 16 were added to each strain culture separately in a 10-mL broth. The heat condition was carried out in a 40°C chamber, with the culture tube sealed completely. Each growth condition was inoculated from the same pre-culture of each strain in a modified YCFAG broth medium, prepared as described in (28). Samples were collected over 24 hours.

### DNA extraction and sequencing following prophage induction

Prophage-induced and naïve *Faecalibacterium* strain cultures were centrifuged for 30 minutes at 4,816 g. Cell pellets were stored at −20°C for subsequent bacterial genomic DNA isolation. The supernatant was filtered twice through 0.45-µm polyethersulfone membrane filters (Sarstedt Inc 83.1826) and formed the virus-like particle (VLP) filtrate. The VLP filtrate was adjusted to a volume of 15 mL using SM buffer (50 mL 1M Tris-HCl pH 7.5, 20 mL 5M NaCl, 8.5 mL 1M MgSO_4_, and 921.5 mL H_2_O) and then concentrated with an Amicon Ultra-15 Centrifugal Filter Unit (100 kDa MWCO) for up to 30 minutes at 4,816 g. The filtrate concentrate was then readjusted to a volume of 15 mL, and the tubes were inverted 30 times to wash the concentrated VLPs with SM buffer. The diluted filtrate was once again concentrated under the same conditions to a volume of approximately 250 µL. Next, 200 µL of the filtrate concentrate was used for nucleic acid extraction after storing at 4°C overnight.

Prior to VLP DNA extraction, cell-free DNA and RNA were removed using enzymatic degradation, for which 200 µL of the filtrate concentrate was mixed with 24 U of TURBO DNase (Invitrogen/Thermo Fisher Scientific), 40 U of RNase I (Thermo Fisher Scientific), and 24 µL 10X DNase buffer (1 mL 100 mM MgCl_2_, Sigma-Aldrich; 1 mL 500 mM CaCl_2_, Sigma-Aldrich; 8 mL H_2_O). This mixture was then incubated at 37°C for 1 hour while shaking at 400 rpm. After that, the mix was incubated at 70°C for 10 minutes to stop the enzymatic reaction. The resulting VLP concentrate was subjected to DNA extraction using the DNeasy Blood & Tissue Kit (Qiagen, Hilden, Germany).

To perform VLP DNA extraction, 180 µL of Buffer ATL and 20 µL proteinase K were added to the VLP concentrate. The mixture was briefly vortexed and incubated at 56°C for 12 minutes while shaking at 400 rpm. Next, 200 µL of Buffer AL was mixed by vortexing with the resulting mix and incubated at 56°C for 10 minutes. After that, 200 µL of ethanol (96–100%) was added, the mixture was vortexed, transferred to the DNeasy Mini spin column, and centrifuged at 8,000 *g* for 1 minute. The mixture was washed with Buffers AW1 and AW2 according to the manufacturer’s instructions. Nucleic acids were eluted by adding 25 µL of Buffer AE to the DNeasy Mini spin column, incubating at 37°C, and centrifuging for 1 minute at 8,000 *g*. The procedure was then repeated with an additional 25 µL of Buffer AE to increase the yield. Double-stranded DNA concentration was determined using the Qubit 1X dsDNA HS kit (Thermo Fisher Scientific).

Microbial DNA was isolated from thawed cell pellets. In preparation for DNA isolation, samples were transferred to a 2-mL screw cap tube containing 0.5 g of 0.1 mm zirconia beads and four 3-mm glass beads. Cell lysis was performed at 3,850 rpm three times for 1 minute, with a 30-second interval, on a Precellys 24 (Bertin Instruments, Montigny-le-Bretonneux, France). Samples were incubated for 15 minutes at 95°C and then centrifuged for 5 minutes at 14,000 rpm and 4°C. About 200 µL of the supernatant was transferred to a new 1.5-mL cup and used for further DNA extraction. The QIAamp DNA mini kit (Qiagen, #51306) was used for DNA extraction in accordance with the manufacturer’s protocol. The following adjustments were made to the protocol: start with 4 µg/mL RNase treatment and incubate for 15 minutes at 37°C. To increase the yield, the volume of elution buffer AE was decreased to 100 µL, and an extra elution step was added.

The extracted DNA was sheared, library-prepped using the NEBNext Ultra II DNA Library Prep Kit, and sequenced on the NovaSeq 6000 platform (Illumina) with 2 × 150 bp paired-end chemistry at Novogene, UK.

### Sequencing read processing

Viral metagenome sequencing data were downloaded from the European Nucleotide Archive, projects PRJNA722819 and PRJNA723467 (254 samples) (29), and PRJNA545408 (130 samples) (30). All sequencing reads were processed using the KneadData 0.10.0 pipeline (https://huttenhower.sph.harvard.edu/kneaddata/). Specifically, reads mapping to the human reference genome GRCh38.p14 (31) were filtered out, and adapter and quality trimming of the reads was performed using Trimmomatic 0.33 (32). KneadData parameters ‘‘--trimmomatic-options "ILLUMINACLIP:NexteraPE-PE.fa:2:30:10:1:true LEADING:20 TRAILING:20 SLIDINGWINDOW:4:20 MINLEN:50" --bypass-trf --reorder’’ were employed. For the PRJNA545408 samples, the adapter file ‘‘TruSeq3-PE-2.fa’’ was used instead. For the samples generated in this study using the NEBNext Ultra II DNA library preparation kit, custom adapters were trimmed by BBMap 38.98 script *bbduk.sh* with the “ktrim=r k=23 mink=11 hdist=1 tpe tbo” parameters, followed by application of KneadData without the “ILLUMINACLIP” specification. Read quality was assessed using FastQC 0.11.9 (33) and MultiQC 1.14 (34).

### Bacterial genome assembly

Assembly was conducted using SPAdes 3.15.5 with the ‘‘--isolate’’ parameter (35). Assembly statistics was calculated using BBMap 39.01 script *statswrapper.sh*.

### Bacterial strain taxonomy assignment

Bacterial strains were assigned to the Unified Human Gastrointestinal Genome (UHGG) collection 2.0.2 (36) species-level clusters based on dereplication with the genomes representing UHGG *Faecalibacterium* clusters by dRep 3.4.2 (37) with the “-pa 0.9 -sa 0.95 -nc 0.30 cm larger” parameters.

### Bacterial gene prediction

Open reading frames (ORFs) in contigs of each bacterial strain were predicted using Prodigal 2.6.3 with the “-p single” parameter (38).

### Prophage prediction by software

Bacterial contigs were screened for prophages using Cenote-Taker2 2.1.5, script *run_cenote-taker2.py* with the “--prune_prophage True --virus_domain_db virion --lin_minimum_hallmark_genes 1” parameters (39), VirSorter2 2.2.4 with the “--include-groups 'dsDNAphage,NCLDV,RNA,ssDNA,lavidaviridae'” parameter (40), and geNomad 1.7.0 (41).

### Sequencing read mapping

Reads were mapped to reference sequences using Bowtie2 2.4.5 (42). Coverage depth and breadth were estimated using the SAMtools 1.17 command *depth* (43) and the BEDTools 2.30.0 command *coverage* (44), respectively. The frequency of nucleotides in the aligned reads at every reference sequence position was assessed using Pysamstats 1.1.2 with the “--type variation --max-depth 100000” parameters (https://github.com/alimanfoo/pysamstats). If >25% of the cumulative length of a *Faecalibacterium* strain contigs was covered by reads from a viral metagenome sample, the sample was considered to be potentially contaminated with *Faecalibacterium* DNA and excluded, with 36 PRJNA722819, 94 PRJNA723467, and 32 PRJNA545408 samples excluded.

### Phage genome assembly

Assembly of VLP reads, obtained following the prophage induction experiments, was conducted using SPAdes 3.15.5 with the ‘‘--metaviral’’ parameter (45). Only contigs recognized as viral by geNomad 1.7.0 (41) were considered.

### Phage packaging mechanism prediction

The packaging mechanisms of phages were predicted by PhageTerm 1.0.12 (46) based on the raw VLP reads obtained following the prophage induction experiments and with a single, prophage-containing, contig submitted using the “--host” parameter. In order to generate split read alignments to analyze the phage Mushu packaging mechanism, VLP reads obtained following the A2-165 prophage induction with 0.1 µg/mL MMC were mapped to the A2-165 genome using BWA-MEM 0.7.17 (47); only the read alignments marked with the tag “SA:Z:NODE_11_” were considered.

### Phage genome comparison to databases

Candidate prophage and phage genomes were compared to 98,780 IMG/VR 4.1 genomes (high-confidence, non-RefSeq, >10 kb, with direct terminal repeats) (3) and 6,395 Viral RefSeq 220 genomes (complete, >10 kb, realm *Riboviria* excluded) (48) using BLASTN 2.14.0+ with the “-task 'blastn' -evalue 0.001” parameters (49). Alignments of cognate genomes, reshuffled so that their 5’-ends match with the help of R package SeqinR 4.2–23 (50), were built using MAFFT 7.505 with the “--nuc --maxiterate 1000” parameters (51).

### Phage gene prediction

ORFs were predicted by Mgcod 1.0.0 with the “--isoforms” parameter (52). Additionally, the “-circular” parameter was used for the Roos, Pioen, Aster, and Lelie phages. All phages and candidate prophages were assigned genetic code 11 by Mgcod.

### Phage protein annotation

The proteomes, predicted as described above, were compared to Pfam 36.0 profiles (53) using the HMMER 3.3.2 program *hmmsearch* with the “--max -E 0.001” parameters (http://hmmer.org/) (54). We only considered protein–profile pairs where ≥100 amino acid residues of the protein were covered by hit(s) to the profile, the profile providing maximal coverage was used for annotation. Coverage was measured in HMMER envelope coordinates, which were combined with the help of R packages rhmmer 0.2.0 and IRanges 2.32.0 (55) in cases of overlap.

### DGR detection

Phage RT genes were annotated based on protein sequence similarity to the Pfam RVT_1 profile, as described above. Multiple sequence alignment (MSA) of the phage RT proteins was constructed by adding their sequences to the Pfam RVT_1 seed MSA using MAFFT 7.505 with the “--amino --add” parameters (51). To detect DGR repeats, each phage genome was compared to itself using BLASTN 2.14.0+ with the “-task 'blastn' -evalue 0.001” parameters (49). A pair of repeats was recognized as DGR repeats if the length of their alignment was ≥90, percent sequence identity ≥50%, and number of mismatches ≥ 5. In addition, ≥75% mismatches were required to correspond to the A nucleotide in one of the sequences (template repeat). DGR repeats were aligned by MAFFT 7.505 with the “--nuc --maxiterate 1000” parameters (51).

### DGR target protein annotation

Sequences of DGR target proteins were compared to each other using the HH-Suite 3.3.0 command *hhalign* with the “-alt 100 -norealign” parameters (56) and to the databases PDB_mmCIF70_24_Dec and Pfam-A_v36 using the HHpred tool with default parameters on the MPI Bioinformatics Toolkit website (4 January 2024) (53, 57, 58). To visualize sequence similarities identified by the latter comparison, the resulting query profiles in the A3M format were downloaded from the MPI Bioinformatics Toolkit website and aligned to the target database profiles using *hhalign* with the “-alt 100 -norealign” parameters.

### Phage taxonomic clustering

Phage genomes were clustered into species-level taxonomic units using the CheckV 1.0.1 script *aniclust.py* with the “--min_ani 95 min_tcov 85 min_qcov 0” parameters (59, 60). Genus-level clustering was conducted by vConTACT2 0.11.3 with the “--db 'ProkaryoticViralRefSeq211-Merged'” parameter (61).

### Phage host prediction

Phage genomes were compared to the 10,969 prokaryotic isolate genomes from the UHGG collection 2.0.2 using BLASTN 2.14.0+ with the “-task 'blastn' -evalue 0.001” parameters (36, 49). A phage was considered to be detected in a prokaryotic isolate if ≥95% of the phage genome length was covered by hits to an isolate contig. In addition, phage genomes were compared to the CRISPR spacers, which were identified in the UHGG isolate genomes by CRISPRidentify 1.2.1 (62), using BLASTN 2.14.0+ with the “-task 'blastn-short' -evalue 1 -max_target_seqs 1000000” parameters. Phages were linked to hosts based on spacer–protospacer matches characterized by ≥95% identity over the length of the spacer.

### Human cohort analysis

Gut metagenomic samples from 1,135 participants of the population-based LifeLines-DEEP (LLD) cohort and 455 patients with inflammatory bowel disease from the 1000IBD cohort were analyzed. The cohort collection, sample processing, and metagenomic sequencing are described in (63, 64). The institutional ethics review board of the University Medical Center Groningen approved the LLD (ref. M12.113965) and the 1000IBD (IRB-number 2008.338) projects. All participants signed an informed consent form prior to sample collection. A phage was considered detected in a sample if ≥75% of its genome length was covered by sample reads (65). Microbial community profiling of the samples was performed using MetaPhlAn 4.1.1 (66), and a microbial taxon was considered detected in a sample if its relative abundance was estimated to be above zero. The prevalence of the five phages and the bacterial genus *Faecalibacterium* in the two cohorts was compared using a logistic regression test. The model was fitted using the R command *glm* with the “family = binomial(link = 'logit')” parameter and was adjusted for age and sex of the cohort participants and the number of clean reads in a metagenomic sample. Benjamini–Hochberg multiple testing correction was conducted using the R command *p.adjust* (67). Relative abundance of the genus *Faecalibacterium* in the two cohorts was compared using Wilcoxon rank-sum test (R command *wilcox.test*).

### Visualization

To display the properties of bacterial (phage) genome sequences, we used a 3,001 (101) nt window sliding along a nucleotide sequence in the 5’ to 3’ direction with a 500 (20) nt step, and the most 3’-terminal window was extended to include up to 1,500 (50) 3’-terminal nucleotides. When a sliding window was applied to an MSA with the help of the R package Bio3d 2.4–4 (68), one of the aligned sequences was designated as a reference, and the MSA columns with reference sequence gaps were excluded from consideration. Similarity of phage genomes was visualized using dot plots: the underlying matching word data were generated using the EMBOSS 6.6.0 command *polydot* with the “-wordsize 12” parameter (69). MSAs were inspected using Jalview 2.11.3.2 (70). RT MSA was visualized with the help of ESPript 3.0 (71).

## RESULTS

### Prophage identification

**Fig 1:**
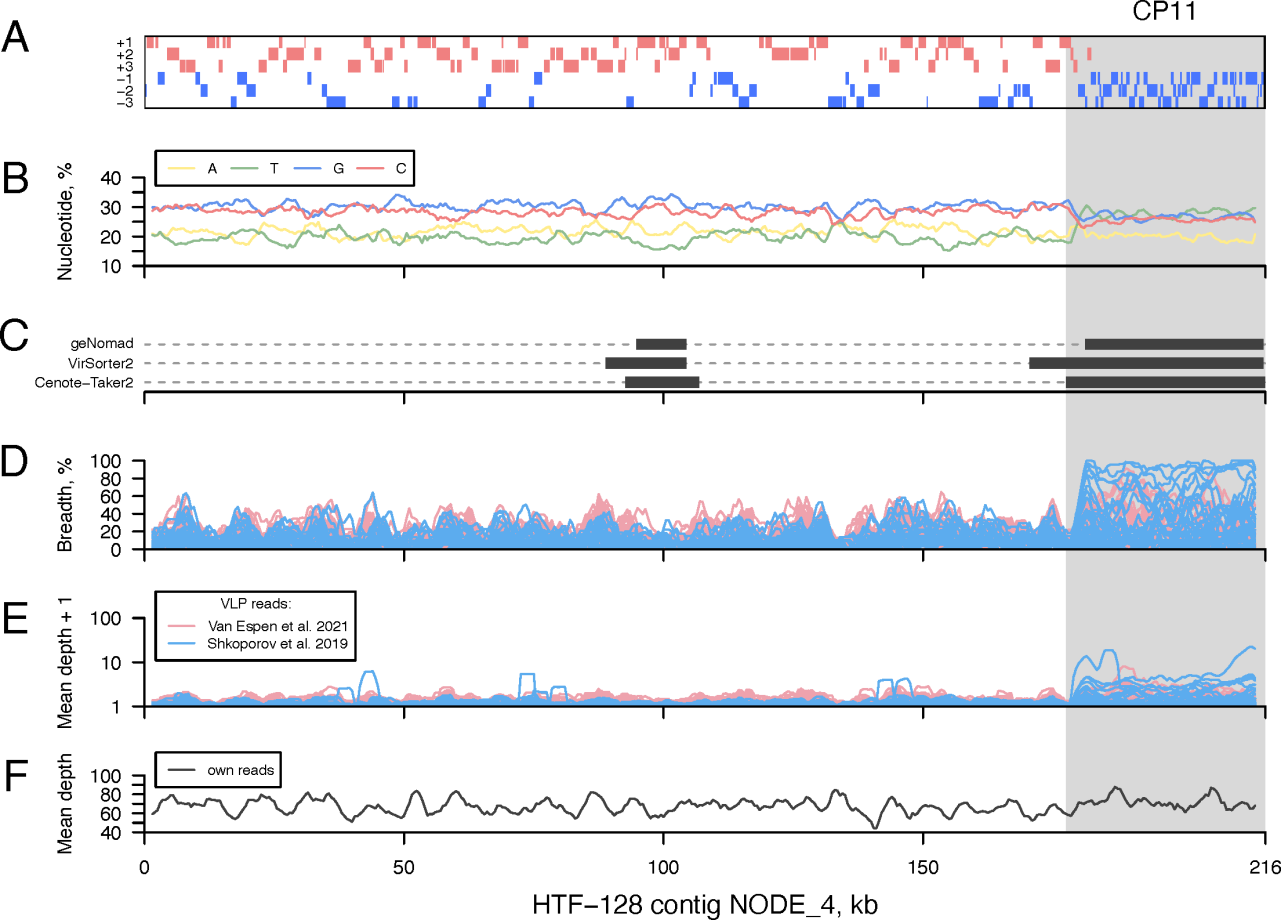
Analysis of bacterial contig properties that can be indicative of prophage presence. The contig NODE_4 of the HTF−128 strain is shown as an example, with the approximate candidate prophage region highlighted by a light gray background. (**A**) ORF organization of the bacterial contig. The contig is shown as a black rectangular contour. ORFs encoded in three positive (negative) reading frames are shown as red (blue) bars. (**B**) Average content of the four nucleotides along the contig. (**C**) Prophage regions of the contig that are predicted by specialized software tools are indicated by dark gray bars. The y-axis indicates the different tools used. (**D**) Breadth and (**E**) depth of contig coverage by viral metagenome reads. Each line corresponds to a sample. Line color indicates the respective study. (**F**) Depth of contig coverage by reads used for bacterial genome assembly. Values on panels B, D–F were recorded using a 3,001-nt window sliding with a 500-nt step. Analysis of all contigs containing candidate prophages is presented in File S2.

To identify prophages, we analyzed the genome assemblies of 22 *Faecalibacterium* strains isolated from human fecal samples (Table S1). Notably, two of the strains, namely, A2-165 and L2-6, were previously demonstrated to harbor prophages by Cornuault *et al*. (21). Bacterial contigs longer than 50 kb were screened for the following indicators of prophage presence. First, compared to bacteria, prophages tend to encode shorter genes with shorter intergenic spaces, and they rarely switch the coding strand (Fig. 1A) (72, 73). Second, the nucleotide content of prophage genomes can differ from that of their bacterial hosts (Fig. 1B) (74). Third, prophages can be detected by specialized software, designed to identify genome sequence regions enriched in phage-specific genes and genome features, for example, geNomad, VirSorter2, and Cenote-Taker2 (Fig. 1C) (39–41). Fourth, prophage genomic sequences are likely to recruit reads (i.e., constitute a reference sequence suitable for read mapping) from publicly available viral metagenomes generated by sequencing VLP DNA isolated from human fecal samples, resulting in elevated breadth and depth of VLP read coverage (Fig. 1D and E) (29, 30). Of note, bacterial DNA transduction or contamination may result in elevated breadth and depth of VLP read coverage as well. Finally, in some cases, prophages may undergo spontaneous induction in a significant portion of the bacterial population, resulting in an elevated amount of the genetic material corresponding to the prophage region (was not observed in the example presented in Fig. 1F) (75). By visually combining these five lines of evidence (Fig. 1), we identified 15 candidate prophages (CPs) in 11 bacterial strains (Table 1; File S1 and S2).

**TABLE 1:** Candidate prophages

Candidate prophage	Host		Location on the host genome	
	Strain	Species-level UHGG cluster	Contig	Coordinates (nt)
CP1^*a*^	FM6	F. prausnitzii	NODE_7	116,041–161,450
CP2	A2-165	F. prausnitzii C	NODE_3	304,537–352,748
CP3	A2-165	F. prausnitzii C	NODE_11	53,400–90,035
CP4	FM8	F. prausnitzii C	NODE_3	52,497–89,308
CP5	HTF-162	F. prausnitzii C	NODE_1	101,208–140,285
CP6	HTF-162	F. prausnitzii C	NODE_2	1–59,981
CP7	HTF-238	F. prausnitzii F	NODE_1	185,626–246,122
CP8^*a*^	HTF-238	F. prausnitzii F	NODE_2	260,693–312,350
CP9	HTF-238	F. prausnitzii F	NODE_5	54,677–87,107
CP10	L2-61	F. prausnitzii G	NODE_9	46,130–99,634
CP11	HTF-128	F. sp900539885	NODE_4	179,869–215,597
CP12^*a*^	HTF-A	F. sp900539885	NODE_1	315,309–370,595
CP13^*a*^	HTF-B	F. sp900539885	NODE_1	144,631–199,917
CP14^*a*^	HTF-C	F. sp900539885	NODE_5	4,667–59,953
CP15^*a*^	HTF-F	F. sp900539885	NODE_4	137,492–192,778

^*a*^ Six CPs that fell into the same viral species-level cluster were represented by CP8 in all subsequent analyses.

We compared the identified CPs to complete phage genomes from the Viral RefSeq and IMG/VR databases (3, 48). Similar phage genomes (>30% of the approximate CP region covered by BLASTN hits to a database genome, see *Materials and Methods* for details) were identified for each of the 15 CPs, providing additional evidence of their phage origin and allowing us to more precisely predict their coordinates on bacterial contigs (Fig. S1). Identification of potential viral structural proteins in the proteome of each CP further pointed to their phage origin (Fig. S2). A comparison of the CP genomes to each other revealed that six CPs belong to the same species-level cluster (Table 1). These six CPs were represented by CP8 in all subsequent analyses, including the induction experiments.

### Prophage induction

To investigate if the identified prophages are able to be induced and start actively replicating, cultures of six *Faecalibacterium* strains that carry 10 CPs were subjected to MMC in concentrations ranging from 0.1 to 16 µg/mL, or to heat treatment (incubation at 40°C), or were incubated without any treatment. Bacterial DNA was isolated from the centrifugation pellet, and viral DNA was isolated from filtered VLPs with the intention to conduct DNA sequencing. However, as the DNA concentrations in most samples were critically low (Table S2), only three samples per strain were selected for sequencing. The only exception was the FM8 strain, for which all eight samples that passed the library quality control filter were sequenced.

**Fig 2:**
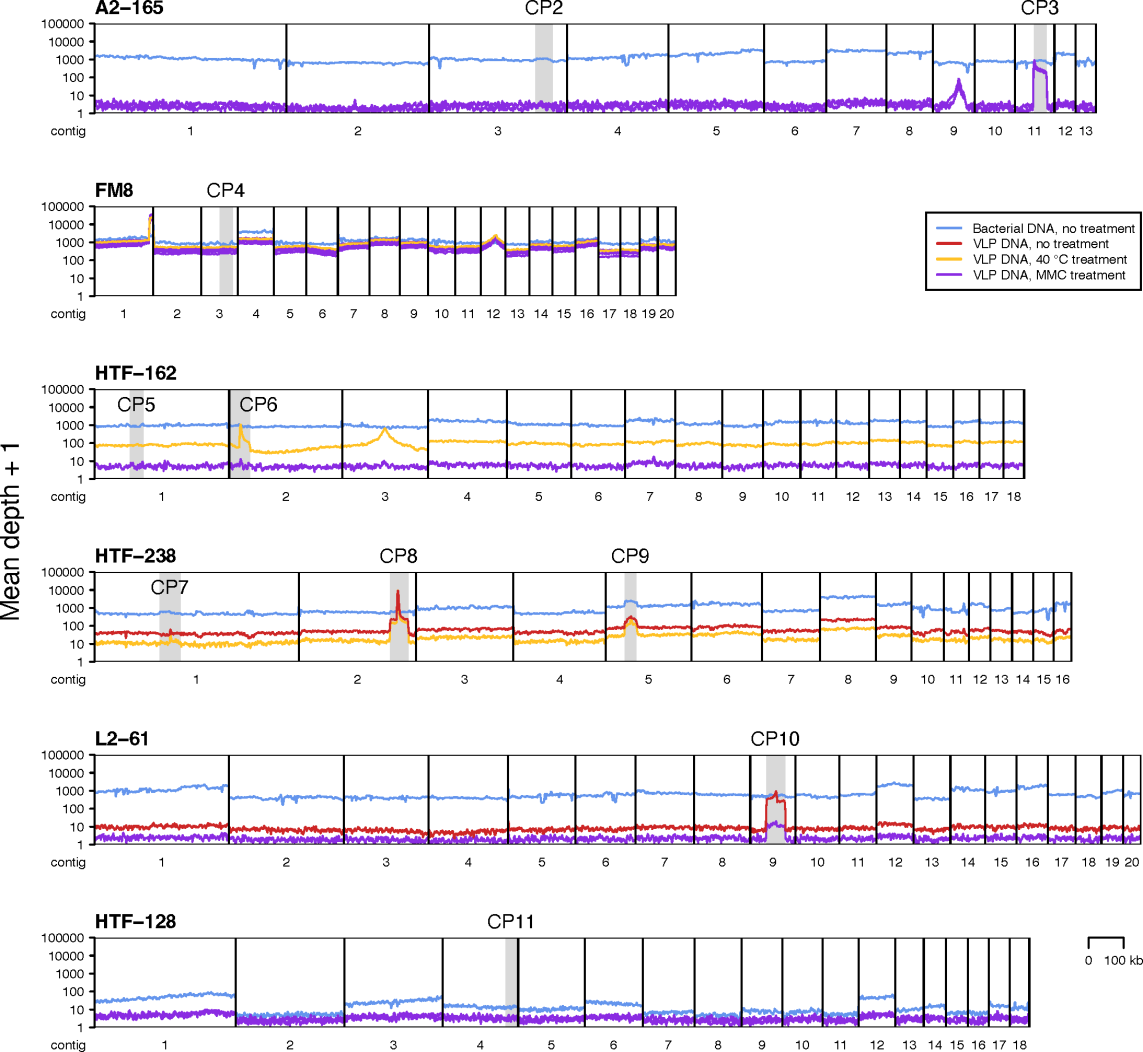
Coverage of bacterial contigs by sequencing reads obtained following prophage induction experiments. Each row corresponds to a *Faecalibacterium* strain (strain names in bold). Each black frame corresponds to a bacterial contig (>50 kb, ordered by size). Each light gray rectangle indicates the position of a candidate prophage. Colored lines depict the depth of contig coverage by sequencing reads from the different DNA fractions (bacterial or VLP DNA) obtained after different prophage induction treatments. See Table S2 for MMC concentrations. Depth of coverage was recorded using a 3,001-nt window sliding with a 500-nt step.

Reads were then mapped back to the bacterial contigs, revealing a pattern indicative of active replication for CP3, CP8, CP9, and CP10, specifically a sharp increase in the number of mapped VLP reads over the prophage region (Fig. 2). In addition, we observed a similar pattern at the 3’-end of the largest FM8 strain contig, consistent with active replication of an additional prophage. In all five cases, there was an indication of prophage activity in every available VLP sample, regardless if MMC, heat, or no induction method was used. The ratio between the mean VLP read coverage depth over a prophage region and over the rest of the bacterial contig containing the prophage varied from 163 to 382 for CP3, from 19 to 56 for CP8, from three to four for CP9, from 11 to 68 for CP10, and from 14 to 47 for the FM8 prophage. Moreover, a complete phage genome matching the prophage region was assembled in all five cases (Table 2). Following the removal of flanking regions (see below), the phage genome corresponding to the CP3 region was found to be identical to that of the *Faecalibacterium* phage Mushu, which was discovered, characterized, and demonstrated to undergo spontaneous induction in the A2-165 strain by Cornuault *et al*. (21). We named the four other phages Roos, Pioen, Aster, and Lelie after the Dutch words for different flowers (Table 2). The assembled genome sequences of phages Roos, Pioen, Aster, and Lelie possessed direct terminal repeats (DTRs).

**TABLE 2:** Active phages

Phage	Candidate prophage	Location on host genome			VLP samples yielding genome assembly	Genome length (nt)	Predicted packaging strategy
		Strain	Contig	Coordinates (nt)			
Mushu	CP3	A2-165	NODE_11	53,400–90,035	0.1 and 2 µg/mL MMC treatment	36,636	Transposable phage
Roos	^*a*^-	FM8	NODE_1	157,269–167,466	40°C and 0.1, 6, 8, 12, and 16 µg/mL MMC treatment	54,314	Headful packaging
			NODE_24	1–42,926			
Pioen	CP8	HTF-238	NODE_2	260,693–312,608	40°C treatment	51,920	Headful packaging
Aster	CP9	HTF-238	NODE_5	54,288–89,871	40°C treatment	35,473	Cohesive ends with 3’-end overhangs
Lelie	CP10	L2-61	NODE_9	46,195–99,602	No treatment	53,400	Cohesive ends with 5’-end overhangs

^*a*^ -, Not applicable.

No other viral genomes were assembled. Although we observed a pattern suggesting that a prophage corresponding to the 3’-terminal part of the CP6 region may have been actively replicating under the 40°C heat treatment (Fig. 2), no matching phage genome was assembled. Interestingly, triangular peaks in VLP read coverage depth (but not bacterial read coverage depth) were associated with A2-165 contig 9, FM8 contig 12, and HTF−162 contig 3 (Fig. 2). These may be indicative of host DNA transduction by virus or virus-like particles (76, 77).

To predict the packaging strategy of the active phages, we used the PhageTerm tool (46). The phages Roos and Pioen were predicted to employ the headful packaging mechanism. Phage Aster was predicted to form cohesive genome ends with 3’-end overhangs, and phage Lelie was predicted to form cohesive genome ends with 5’-end overhangs. Finally, phage Mushu was predicted to replicate via transposition and to package a genome flanked by variable host genome fragments excised from the transposition sites (Table 2), consistent with the results of the study by Cornuault *et al*. (21).

In order to predict the exact termini of the Mushu genome assembled from VLP reads in our study, we mapped VLP reads, obtained following the A2-165 prophage induction with 0.1 µg/mL MMC, to the A2-165 genome assembly using the BWA-MEM software (47). There were 2,599 split read alignments linking distant A2-165 genome fragments to the A2-165 contig containing Mushu in prophage form. The majority of these alignments supported the link to two specific nucleotides at the borders of the prophage region (Fig. S3A and B), consistent with termini of a transposable phage. This analysis also indicated that Mushu transposition sites are located throughout the host genome (Fig. S3C).

Assembling complete, terminally redundant genomes of the phages Roos, Pioen, Aster, and Lelie and mapping them back to the bacterial contigs containing the respective prophages (Table 2) allowed us to identify phage integration sites with high precision. The integration sites of the four phages appear to be positioned within bacterial intergenic regions. Notably, phages Roos and Aster each encode an *Escherichia* phage λ integrase homolog (78), while phages Pioen and Lelie each encode a protein that shares homology with the *Streptomyces* phage φC31 integrase (79).

The variable host genome fragments at the termini of the assembled Mushu genome were removed, resulting in a genome identical to the one assembled by Cornuault *et al*. (21). The assembled genome sequences of phages Roos, Pioen, Aster, and Lelie were rearranged by PhageTerm to make the sequence start at the predicted *pac* or *cos* site, consistent with a packaged genome. Interestingly, when sequencing reads were mapped to the resulting genome sequences (Fig. S4), the number of mapped VLP reads was elevated at the start of each genome. This pattern may reflect the presence of DTRs in packaged Roos and Pioen, or, possibly, partial release of genome molecules from the capsids.

### Active phages show similarity to known phages

**Fig 3:**
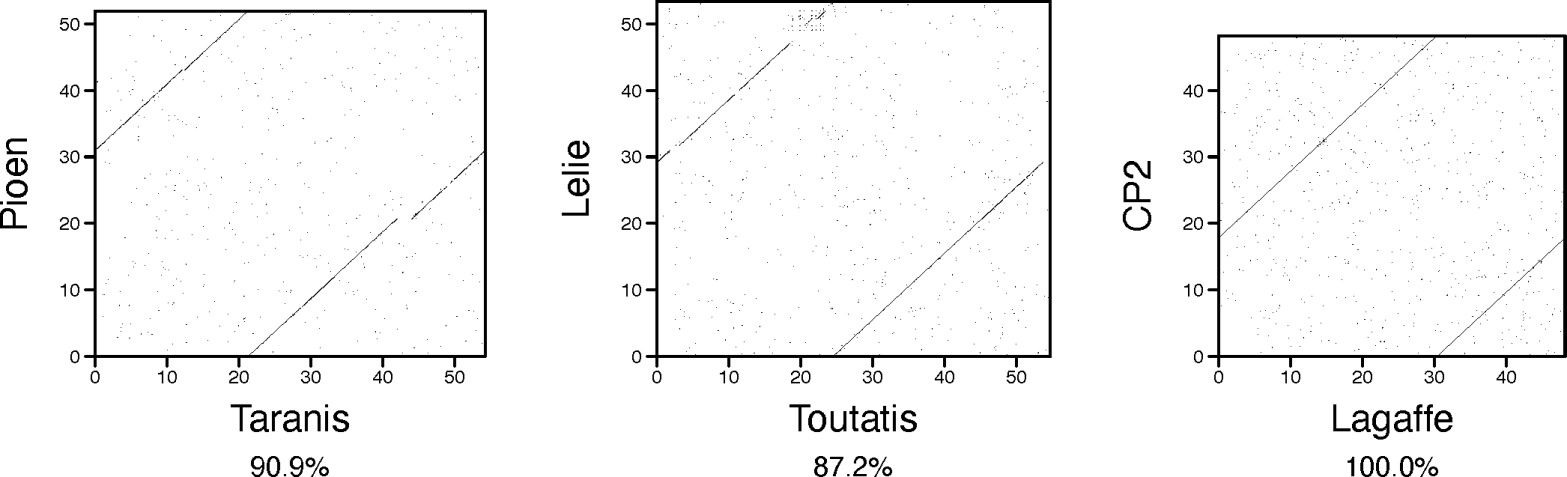
Similarity between the known phages and phages detected in this study. Similarity between pairs of genomes assigned to the same genus-level cluster, illustrated by dot plots. X-axis of each dot plot corresponds to a phage described in the literature. Y-axis corresponds to a phage or CP detected in this study. Coordinates are indicated in kilobases. Every 12-letter word (i.e., a 12-nucleotide block) shared by a pair of sequences is presented as a black dot on a dot plot. The percent of columns with identical nucleotides in a pairwise alignment of the two sequences is given below each dot plot.

To analyze similarities between the active phages and CPs detected in this study and previously described phages, we built a vConTACT2 gene-sharing network that included the prokaryotic virus fraction of the Viral RefSeq database (61). Within the network, all the active phages and CPs we detected fell into the connected components of the graph populated by viruses belonging to class *Caudoviricetes*. Besides the phage Mushu, two active phages were assigned to vConTACT2 genus-level clusters harboring genomes from Viral RefSeq. Specifically, phages Pioen and Lelie were each assigned to a cluster with a *Faecalibacterium* prophage described by Cornuault *et al*.: Taranis and Toutatis, respectively (21). Genomes of Pioen and Taranis displayed 90.9% nucleotide identity, and genomes of Lelie and Toutatis, 87.2% nucleotide identity (Fig. 3). Notably, CP2, which failed to be induced in our study, was also assigned to a genus-level cluster with a previously described phage, namely, phage Lagaffe discovered, characterized, and demonstrated to undergo spontaneous induction in the A2-165 strain by Cornuault *et al*. (21). CP2 was found to differ from Lagaffe by a single-nucleotide insertion (a guanine insertion after the Lagaffe nucleotide 41,194) and is further referred to as Lagaffe_CP (Fig. 3).

### Active phages are rich in diversity-generating retroelements

**Fig 4:**
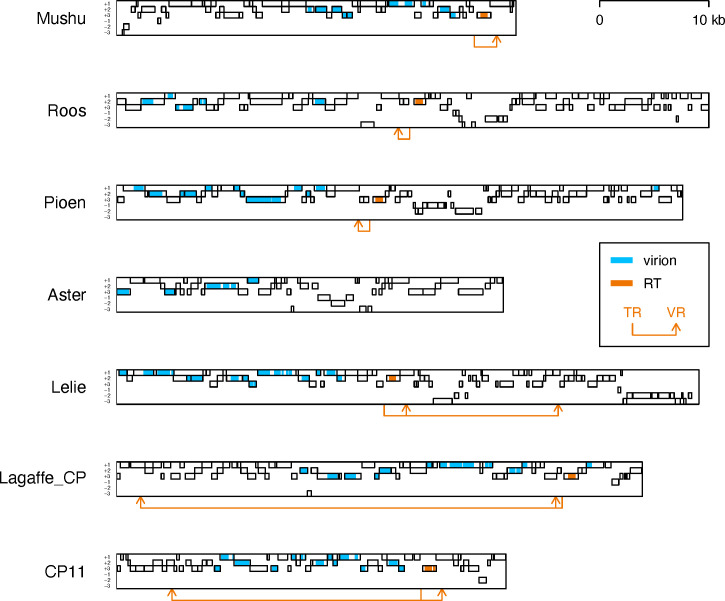
Genome maps of the active phages and DGR-containing candidate prophages. Each genome is depicted as a black rectangular contour. ORFs encoded in three positive and three negative reading frames are shown as light gray bars. Regions of ORFs matching Pfam profiles of virus structural proteins, as well as proteins involved in virus DNA packaging and virion assembly, are highlighted in blue. Regions of ORFs matching the Pfam RVT_1 profile are highlighted in orange. The orange arrows point from a DGR template repeat to a DGR variable repeat location.

Analysis of the genomes of active phages showed that four of the five phages (Mushu, Roos, Pioen, and Lelie) possess key components of a DGR (Fig. 4): an RT gene (Fig. S5) and characteristic nucleotide repeats (Fig. S6; Table S3). The DGR of Mushu was previously described by Cornuault *et al*. (21). The overall observed richness in DGRs is also in line with the findings of the study by Cornuault *et al.*, who reported that a high proportion of *Faecalibacterium prausnitzii* phages possess DGRs (21).

Interestingly, we identified two variable regions in the genome of phage Lelie. Furthermore, when inspecting the CPs that failed to be induced in our study, two of the genomes, Lagaffe_CP and CP11, were found to possess a DGR, and the DGR included two variable regions in both cases (Fig. 4). Importantly, the DGR of phage Lagaffe was identified previously, although only a single variable region was described in the original study (21).

**Fig 5:**
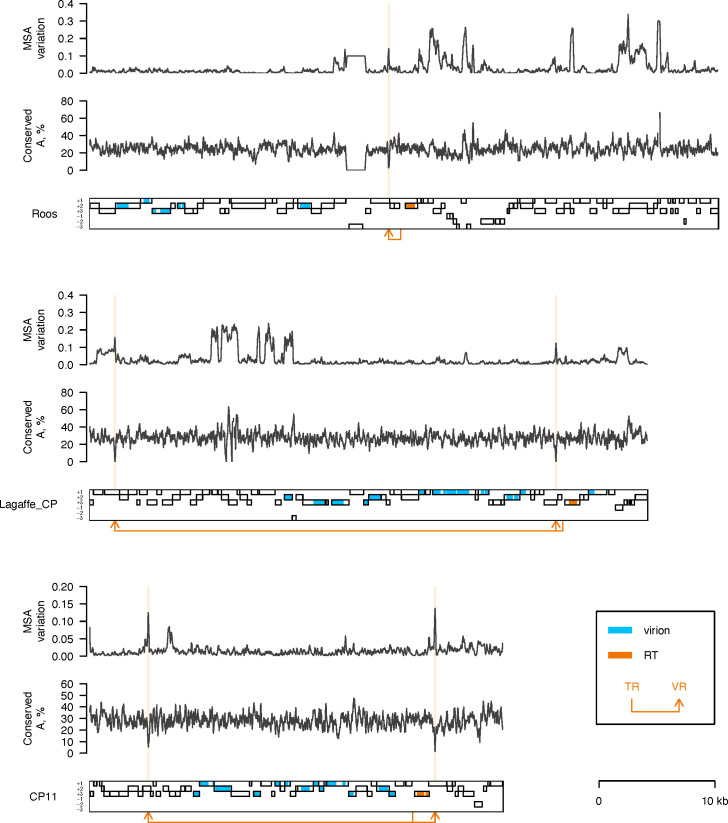
Nucleotide variation and adenine conservation in multiple sequence alignments of cognate phage genomes. Data for the phage Roos, Lagaffe_CP, and CP11 are presented above their genome maps (see Fig. 4 legend for details). Locations of variable regions are highlighted by light orange vertical lines. Nucleotide variation in an MSA column was calculated as a proportion of symbols different from the most frequent one and subsequently averaged using a 101-nt window sliding with a 20-nt step. Adenine conservation was estimated as the percent of conserved adenine columns among all conserved columns in a 101-nt sliding window. A conserved MSA column was defined as a column in which the most frequent symbol occupied ≥90% positions.

To verify that the identified DGR elements can be active, we analyzed nucleotide variation and adenine conservation in alignments of cognate phage genomes. The genomes of the DGR-containing phages and CPs were compared to the Viral RefSeq and IMG/VR databases in order to identify cognate phage genomes (≥95% query genome length coverage by BLASTN hits to a database genome, see *Materials and Methods* for details). Multiple cognate genomes were identified for phage Roos (*n* = 9), Lagaffe_CP (*n* = 18), and CP11 (*n* = 37), allowing us to build MSAs based on their genomes and analyze nucleotide variation and adenine conservation in these MSAs (Fig. 5). In all three cases, there was a notable spike in nucleotide variation and a decline in adenine conservation at the variable regions, which is consistent with adenine-specific hypermutation induced by a DGR. Importantly, the signal was observed at both variable regions of Lagaffe_CP and CP11 (Fig. 5).

**Fig 6:**
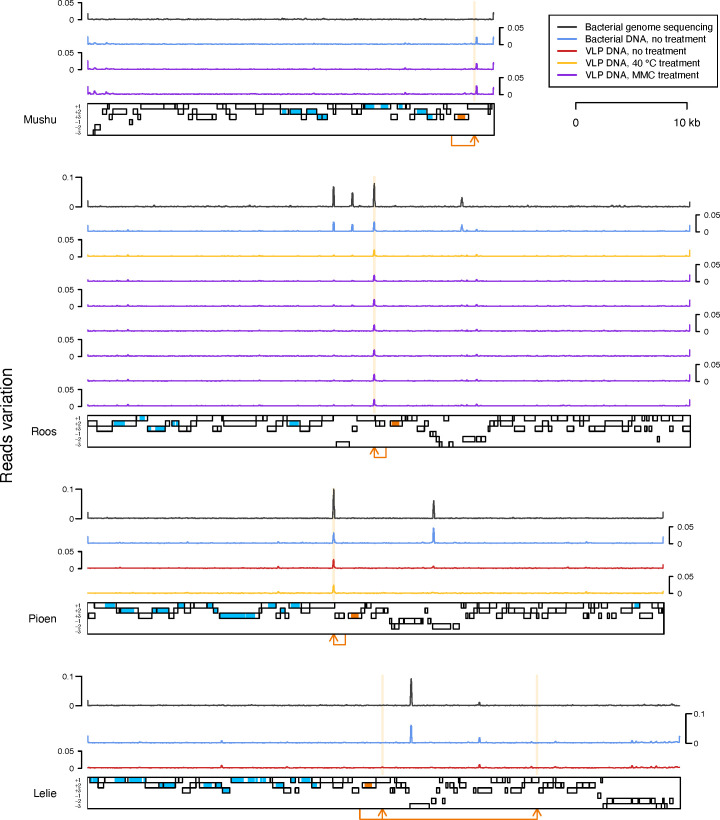
Nucleotide variation in sequencing reads mapped to phage genomes. Data for the four DGR-containing phages are shown above their genome maps (see Fig. 4 legend for details). Locations of variable regions are highlighted by light orange vertical lines. Information about each sequencing sample originating from the host culture of a phage is presented on a separate line. They are shown in black for the initial bacterial genome sequencing and in color for sequencing following prophage induction experiments (see legend for colors). Only samples with coverage depth ≥10 along ≥95% phage genome length are shown. The nucleotide variation in reads mapped to a genome position was estimated as a proportion of nucleotides different from the most frequent one and subsequently averaged using a 101-nt window sliding with a 20-nt step.

Analysis of nucleotide variation in the sequencing reads mapping to the genomes of the DGR-containing phages and CPs offers an alternative approach to capturing DGR activity, which is focused on a (pro)phage at a specific moment in time. To explore this approach, we mapped reads from various samples sequenced in this study (initial bacterial genome sequencing samples and bacterial and VLP DNA samples obtained following the prophage induction experiments) to the respective phage and CP genomes (Fig. 6; Fig. S7). The clearest signal consistent with DGR activity was observed in phages Roos and Pioen, where we detected a nucleotide variation peak at the variable region in all available samples (Fig. 6). For Mushu, no peak at the variable region was observed when analyzing the initial bacterial genome sequencing sample and, although a peak was observed in the three other sequencing experiments involving Mushu, it seems to be shifted to the 3’-end, relative to the variable region (Fig. 6). No peaks were observed at the variable regions of phage Lelie (Fig. 6). Likewise, there were no DGR activity signals in the limited data available for Lagaffe_CP and CP11 (Fig. S7). A lack of signal may indicate that a DGR was inactive at the time of sequencing.

### DGR target proteins of diverse phages display similarity

**Fig 7:**
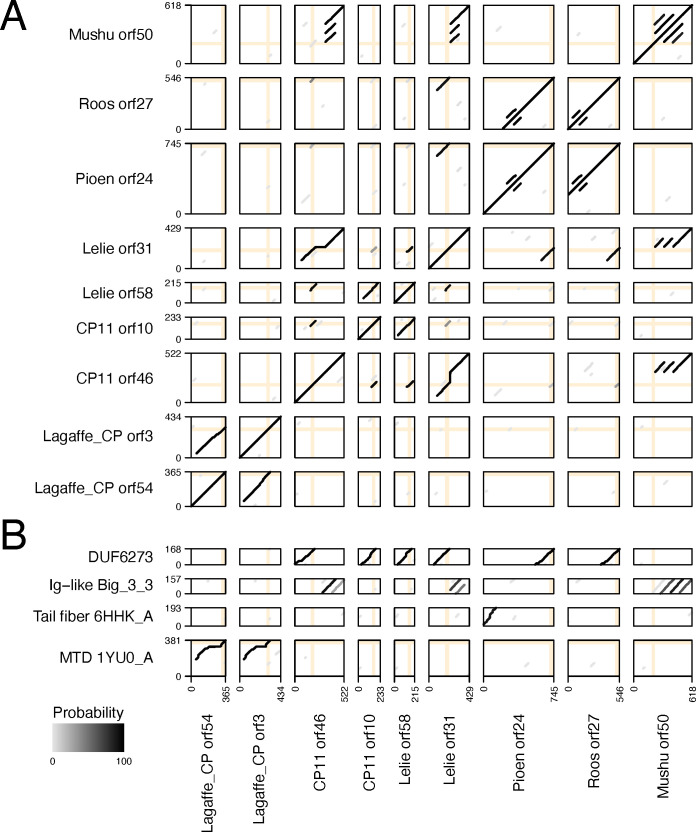
Sequence similarities of the DGR target proteins. Pairwise comparisons between the DGR target proteins and (**A**) themselves and (**B**) selected PDB and Pfam database entries. Each comparison is presented in a frame where the X-axis and Y-axis coordinates correspond to query and target amino acid residues, respectively. Query and target variable regions are designated by orange vertical and horizontal lines, respectively. HHalign alignment paths are shown by gray lines, and the darkness of each line indicates the Probability value assigned to the respective alignment. Panel A alignments are between individual sequences. Panel B alignments are between HHpred-generated query profiles and database entries: DUF6273, Pfam domain of unknown function; Big_3_3, Pfam bacterial immunoglobulin-like domain; 6HHK_A, a profile based on the PDB structure of the *Listeria* phage A511 tail fiber protein gp105; and 1YU0_A, a profile based on the PDB structure of the *Bordetella* phage BPP-1 major tropism determinant.

Functional annotation of the proteins diversified by DGRs is essential for uncovering the role played by the DGR in the virus life cycle. As a first step toward functional characterization of the DGR target proteins, we compared their sequences to each other (Fig. 7A). This comparison revealed that the proteins fall into several sequence similarity groups. The two Lagaffe_CP DGR target proteins displayed strong similarity along a significant portion of their lengths (HHalign Probability 99.9%; Identities 40%). The DGR target proteins of phages Roos and Pioen were nearly identical (Probability 100.0%; Identities 97%), but for an extra N-terminal domain present in the Pioen protein. The large DGR target proteins of Lelie and CP11 were similar (Probability 99.0%; Identities 34%), as were their small DGR target proteins (Probability 97.1%; Identities 33%). DGR target protein similarity in the otherwise divergent phages Pioen and Roos, Lelie and CP11 is suggestive of a similar functional role played by the proteins and their DGR-induced hypermutation.

To shed light on the possible function of the DGR target proteins, we further compared them to Pfam and PDB database entries (53, 57), which yielded several high-scoring hits (Fig. 7B). Consistent with the results of the study by Cornuault *et al*. (21), this comparison revealed that the Mushu DGR target protein includes several tandem repeats of the bacterial immunoglobulin (Ig)-like domain, a domain architecture similar to that of the phage T4 Hoc-like capsid decoration protein implicated in phage adherence to mucus (21, 80–82). Both the large and small DGR target proteins of Lelie and CP11, as well as the DGR target proteins of Roos and Pioen, were shown to include a domain of unknown function named DUF6273, with the variable regions of all these proteins mapping to the C-terminus of DUF6273. Interestingly, the N-terminal domain of the Pioen DGR target protein was found to be similar to the gp105 protein comprising a fraction of the *Listeria* phage A511 tail fiber (83). Finally, both Lagaffe_CP DGR target proteins were found to be similar to the MTD of the *Bordetella* phage BPP-1 (84) and the variable regions of the proteins aligned (Fig. 7B). Importantly, similarity to the BPP-1 MTD was previously reported for the Lagaffe DGR target protein described by Cornuault *et al*. (21).

### Phage ecology

**Fig 8:**
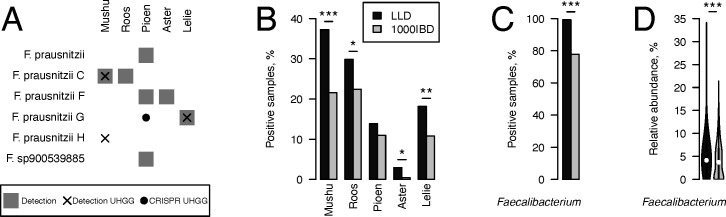
Phage ecology. (**A**) Predicted hosts of the phages. Host UHGG species-level clusters are specified. Hosts were assigned based on the phage detection source in our study (gray square), phage detection in a UHGG isolate (cross), and phage match with a UHGG isolate CRISPR spacer (black circle). (**B**) Prevalence of the phages in the gut metagenomes of Lifelines-DEEP and 1000IBD cohort participants. (**C**) Prevalence and (**D**) relative abundance of the genus *Faecalibacterium* in the gut metagenomes of Lifelines-DEEP and 1000IBD cohort participants. *P*-values on panel B–C are corrected for multiple testing using the Benjamini–Hochberg method. * *P*-values < 0.05, ** *P*-values < 0.01, and *** *P*-values < 0.001.

By comparing the genomes of the five active phages to the genomes and CRISPR spacers of the prokaryotic isolates in the UHGG collection (36), we linked the phages to additional hosts (Fig. 8A). All the predicted hosts belonged to genus *Faecalibacterium*, and, interestingly, Mushu-like and Pioen-like phages were predicted to infect bacteria from more than one UHGG species-level cluster.

*Faecalibacterium* is one of the most common butyrate-producing bacteria in the human gut (85). The decrease in abundance of *Faecalibacterium* species has been consistently associated with gastrointestinal diseases, including inflammatory bowel disease (IBD), irritable bowel syndrome, and colon cancer (86–88), whereas high levels of *Faecalibacterium* are positively associated with general health (89). Its administration reduced inflammation and aberrant crypt formation in multiple *in vivo* and *in vitro* studies (90, 91). We therefore analyzed the prevalence of the five active *Faecalibacterium* phages in human gut metagenomes of individuals from the population cohort LifeLines-DEEP (*n* = 1,135) and the 1000IBD cohort of inflammatory bowel disease patients (*n* = 455) (63, 64). A phage was considered detected (in active or prophage form) in a metagenome if ≥75% of its genome was covered by sample reads. Phages Mushu, Roos, Aster, and Lelie were found to be less prevalent in the metagenomes of 1000IBD cohort participants compared to the general population (*P*-value < 0.05, Fig. 8B), using a linear regression model adjusted for the age and sex of cohort participants and the number of clean reads per sample. Importantly, the decreased phage prevalence may be explained by decreased prevalence of their hosts, bacteria belonging to genus *Faecalibacterium* (*P*-value < 0.05, Fig. 8C). When the model was further adjusted for the log-normalized relative abundance of the genus *Faecalibacterium* (Fig. 8D), associations of Mushu and Aster with IBD diagnosis remained marginally significant (*P*-value = 0.04), while Roos and Lelie associations lost significance (*P*-value > 0.05).

## DISCUSSION

In this study, we developed a bioinformatic approach to prophage identification in bacterial genome assemblies. We utilized this approach to identify 15 CPs in 22 *Faecalibacterium* strains. To further test the approach, we conducted prophage induction experiments with six strains containing 10 CPs. These experiments yielded five active phages: four actively replicating CPs and an additional actively replicating prophage that was not identified by our bioinformatic approach. We further analyzed the identified phages by comparing them with known phages, predicting their genome packaging strategy and characterizing their DGRs. We also investigated the prevalence of these phages in a large population cohort and in patients with IBD.

Importantly, there are several limitations associated with our prophage detection approach and experimental setup. Although our prophage detection approach is unique in that it utilizes publicly available viral metagenomes, analysis of genome sequence properties, and existing prophage-detecting tools geNomad, VirSorter2, and Cenote-Taker2 (39–41), there is a number of other high-quality prophage-detecting tools that were not utilized in our study, for example, PHASTEST and PhiSpy (92, 93). When detecting prophages, we only considered bacterial contigs longer than 50 kb. This, along with the fragmentation of bacterial genome assemblies, may negatively affect the number of detected prophages, as illustrated by our failure to detect candidate prophage corresponding to the active phage Roos. Low DNA concentration in our VLP DNA sequencing samples prevented sequencing of several samples and may preclude detection of low prophage and DGR activity in the sequenced samples. Increasing the volume of bacterial cultures used in induction experiments and optimizing induction conditions can help solve these limitations in future studies. The available sequencing data did not allow us to determine if a prophage had undergone spontaneous induction or was induced as a result of MMC or heat treatment in each case. In future studies, in addition to VLP DNA sequencing, PCR designed to detect products of prophage excision and circularization can be used to access prophage activity under non-inducing and inducing conditions (94).

Interestingly, the genomes of two active phages observed in our study, Roos and Aster, appear to belong to genera not yet represented in Viral RefSeq. In contrast, phage Mushu was described previously (21, 95, 96), while the genomes of Pioen and Lelie fell into genus-level clusters containing the previously described *Faecalibacterium* prophages Taranis and Toutatis, respectively (21). Unlike Mushu, which had been shown to undergo spontaneous induction, the activity of Taranis and Toutatis had not been tested at the time of their discovery (21). Consequently, activity of the related prophages Pioen and Lelie demonstrated in this study offers additional confirmation of activity for the respective prophage clusters.

Notably, six CPs failed to be induced in our experimental setup. There are several possible explanations for this. First, a prophage can lose its ability to enter a lytic cycle and become cryptic (97). A cryptic prophage may retain a degree of genome similarity to related phages and recruit reads from viral metagenomes, but it would be impossible to induce it. Second, the induction conditions we applied might not have been suitable to activate an otherwise functional prophage. This appears likely for Lagaffe_CP and CP11, as we identified a large number of highly similar phage genomes with DTRs in databases, and as phage Lagaffe, the genome of which differs from that of Lagaffe_CP by a single-nucleotide insertion, had been demonstrated to undergo spontaneous induction (21). Finally, a prophage may be a satellite and depend on proteins of a helper phage to produce phage particles (98).

One of the most remarkable features of the phages Mushu, Roos, Pioen, and Lelie, as well as Lagaffe_CP and CP11, is the presence of the key DGR components in their genomes, specifically a TR, VR, and an RT gene (21). Moreover, Lelie, Lagaffe_CP, and CP11 possess a second VR.

The exact function of the DGR target proteins analyzed in this study is unknown, but their domain organization suggests that some can be structural proteins. For example, the domain organization of the Mushu target protein resembles that of the phage T4 Hoc-like capsid decoration protein implicated in phage adherence to mucus (21, 80–82), while the N-terminal domain of the Pioen target protein is similar to the tail fiber protein of the *Listeria* phage A511 (83). The interactions between phage structural proteins and molecules found in the environment and on the surface of host cells are essential for phage dissemination and host cell entry, and DGR may help adapt structural proteins for interactions with a changing environment or host.

The function of the pairs of target proteins found in Lelie, Lagaffe_CP, and CP11 is even more intriguing. Lagaffe_CP encodes two target proteins homologous to MTD of the *Bordetella* phage BPP-1 (84). Consequently, it is tempting to suggest that DGR may broaden the host range of Lagaffe_CP through a mechanism similar to that of the BPP-1 phage, doubling its efficiency due to the presence of two homologous target proteins. Lelie and CP11 each encode a pair of target proteins whose function is not entirely clear. Possibly, the two proteins are involved in different aspects of the phage–host or phage–environment interactions, and the DGR-induced hypermutation allows optimization of both. Notably, DGR diversification of two different phage proteins, a predicted tail protein and a protein of unknown function, had been reported in *Roseburia* phage Shimadzu (18).

One of the lesser studied aspects of the DGR mechanism is the timing of DGR activity. Is DGR activity constant? Can changes in the environment or events in the phage life cycle trigger DGR activation? Our data provide some interesting clues. At the level of a group of cognate phage genomes, we observed a pattern of nucleotide variation consistent with DGR activity within every group we were able to analyze (Roos, Lagaffe_CP, and CP11 groups). But at the level of individual phages, sequencing read variation at the VRs was observed only for Roos and Pioen. It can be suggested that the DGRs of Mushu, Lelie, Lagaffe_CP, and CP11 found in our bacterial cultures are not functional, but temporary inactivation seems more likely. The fact that DGR activity had been reported in phages Mushu and Lagaffe (21) seems to support the temporary inactivation hypothesis.

Overall, our study shows that identification and experimental characterization of prophages integrated into the genomes of cultured gut bacteria can further our understanding of phage phenomena like targeted hypermutation by DGR.

## Data Availability

The code used to conduct the analysis was deposited to the GitHub repository https://github.com/aag1/Faecalibacterium_prophages/. Raw sequencing data were deposited to the NCBI Sequence Read Archive under BioProject PRJNA1082652. Genomes of the phages Roos, Pioen, Aster, and Lelie were deposited to the NCBI GenBank under the accession numbers PP479939–PP479942.

## SUPPLEMENTAL MATERIAL

Supplemental figures spectrum.01066-24-s0001.pdf Fig. S1 to S7.

Supplemental file S1 spectrum.01066-24-s0002.txt Sequences of the bacterial contigs containing candidate prophages.

Supplemental file S2 spectrum.01066-24-s0003.pdf Analysis of the bacterial contigs containing candidate prophages.

Supplemental tables spectrum.01066-24-s0004.xlsx Tables S1 to S3.

ASM does not own the copyrights to Supplemental Material that may be linked to, or accessed through, an article. The authors have granted ASM a non-exclusive, world-wide license to publish the Supplemental Material files. Please contact the corresponding author directly for reuse.
