# Supplementary material for: Identification and characterization of Faecalibacterium prophages rich in diversity-generating retroelements
Source: Microbiol Spectr. 2024 Dec 31;13(2):e01066-24. doi: 10.1128/spectrum.01066-24 (PMC11792537; doi:10.1128/spectrum.01066-24)
Supplement: Supplemental figures — Fig. S1 to S7. [file spectrum.01066-24-s0001.pdf]

SUPPLEMENTARY FIGURES

Identification and characterization of *Faecalibacterium* prophages rich in diversity-generating retroelements

Anastasia Gulyaeva[1*], Lei Liu[2], Sanzhima Garmaeva[1], Marloes Kruk[1], Rinse K. Weersma[1,3], Hermie J. M. Harmsen[2], Alexandra Zhernakova[1*]

[1] Department of Genetics, University Medical Center Groningen, Groningen, the Netherlands
[2] Department of Medical Microbiology, University Medical Center Groningen, Groningen, the Netherlands
[3] Department of Gastroenterology and Hepatology, University Medical Center Groningen, Groningen, the Netherlands
[*]Correspondence should be addressed to A.G. (a.gulyaeva@umcg.nl) and A.Z. (a.zhernakova@umcg.nl).

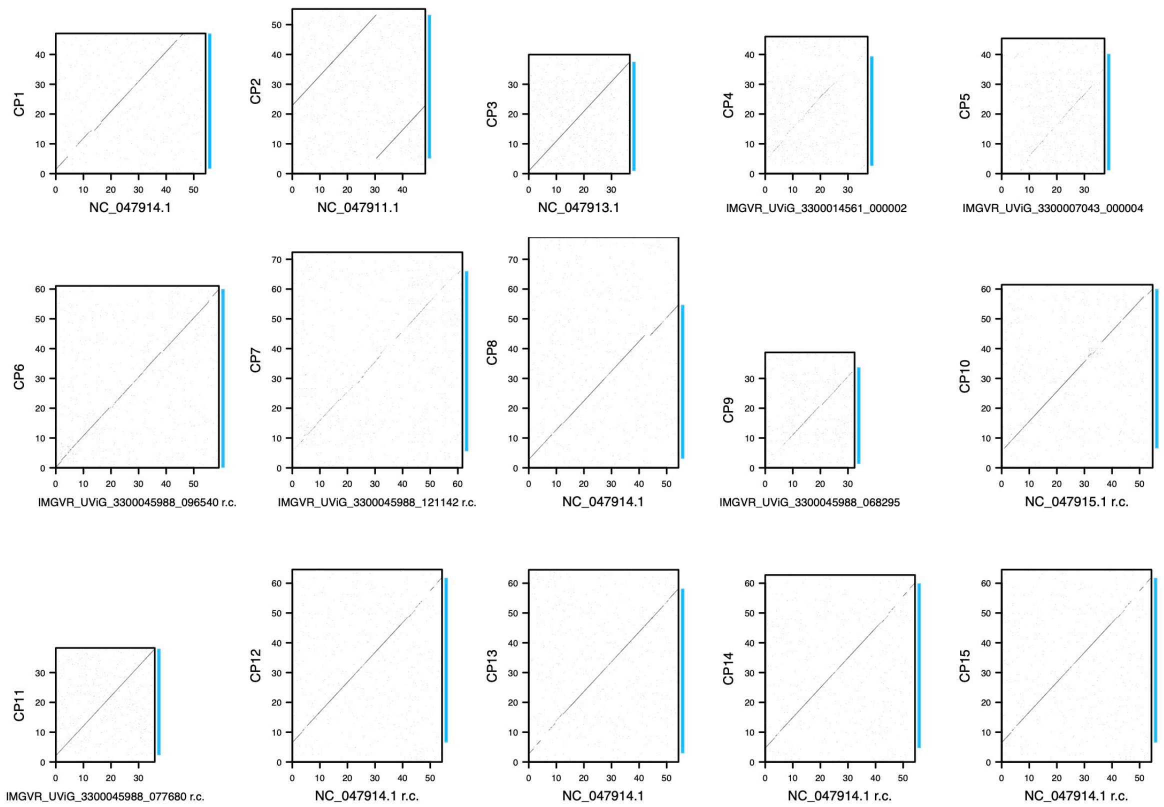

Figure S1. Sequence similarity between the candidate prophage genomes and complete phage genomes from the databases. Each dot plot illustrates the similarity between a pair of sequences. X-axis corresponds to a complete phage genome from the RefSeq or IMG/VR database. Y-axis corresponds to a candidate prophage. Coordinates are indicated in kilobases. Every 12-letter word (i.e., a 12-nucleotide block) shared by a pair of sequences is shown as a black dot on a dot plot. If a reverse complement of a sequence was utilized, the letters “r.c.” are added to the sequence identifier. Refined coordinates of the candidate prophages are indicated by vertical blue lines.

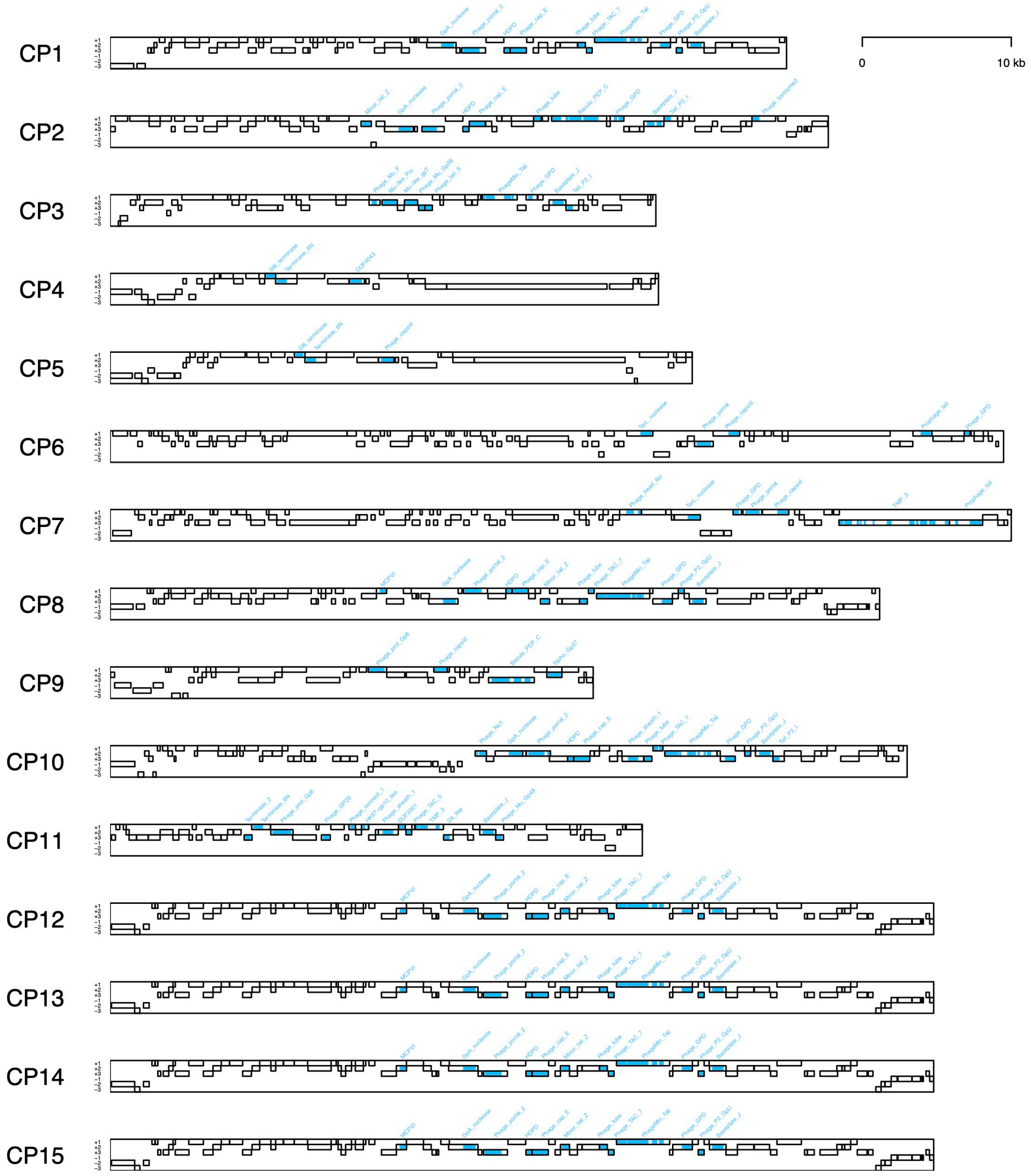

Figure S2. Genome organization of candidate prophages. Each genome is depicted as a black rectangular contour. In cases where most ORFs were encoded on the negative strand of the original sequence, its reverse complement is presented. ORFs encoded in three positive and three negative reading frames are shown as light gray bars. Regions of ORFs matching Pfam profiles of virus structural proteins, as well as proteins involved in virus DNA packaging and virion assembly, are highlighted in blue. The names of the profiles are indicated above.

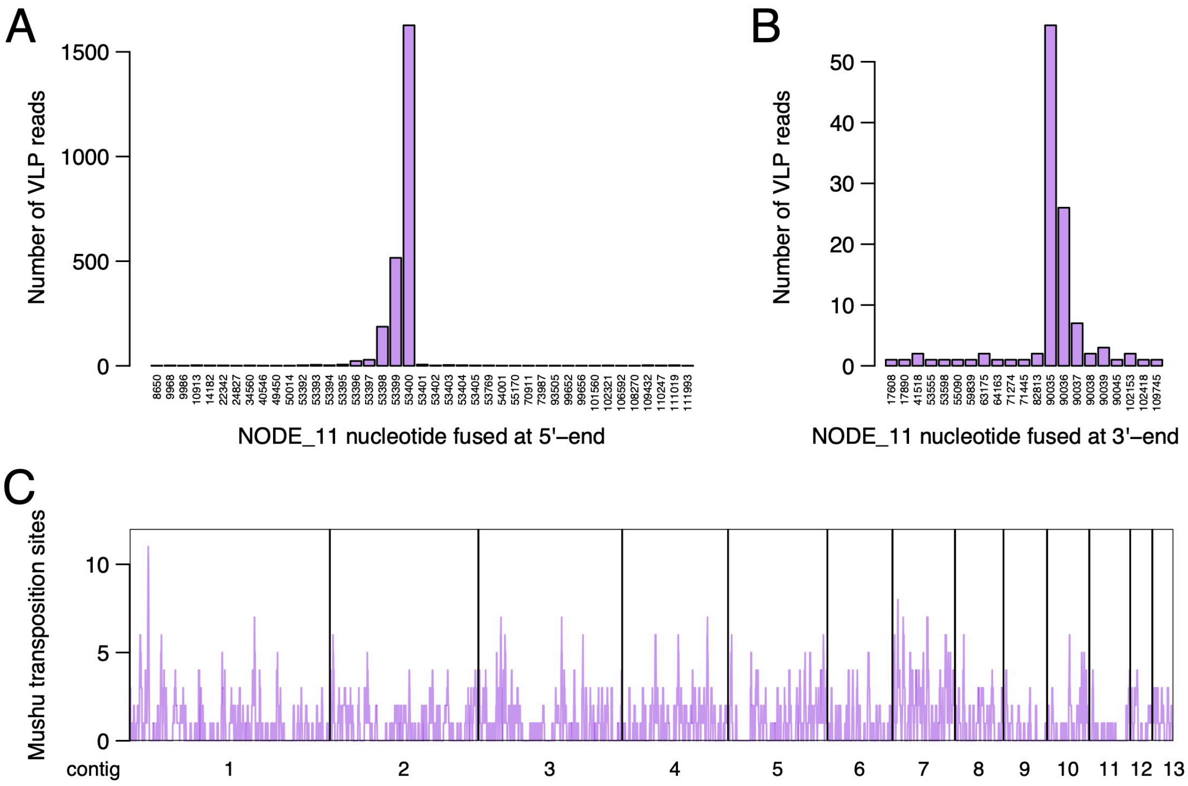

Figure S3. Phage Mushu genome termini and transposition sites. Data is based on split read alignments reported by the BWA-MEM software when mapping VLP reads, obtained following the A2-165 prophage induction with 0.1 µg/ml MMC, to the A2-165 genome assembly. Nucleotides of the Mushu-containing contig NODE_11, fused to distant A2-165 genome fragments at the (**A**) 5'-end and (**B**) 3'-end, are shown, along with the number of read alignments supporting the fusion. (**C**) Number of Mushu transposition sites along the A2-165 contigs >50 kb is displayed using a 3,001 nt window sliding with a 500 nt step. The transposition sites were identified based on read alignments connecting A2-165 genome fragments to the nucleotide 53,400 of NODE_11.

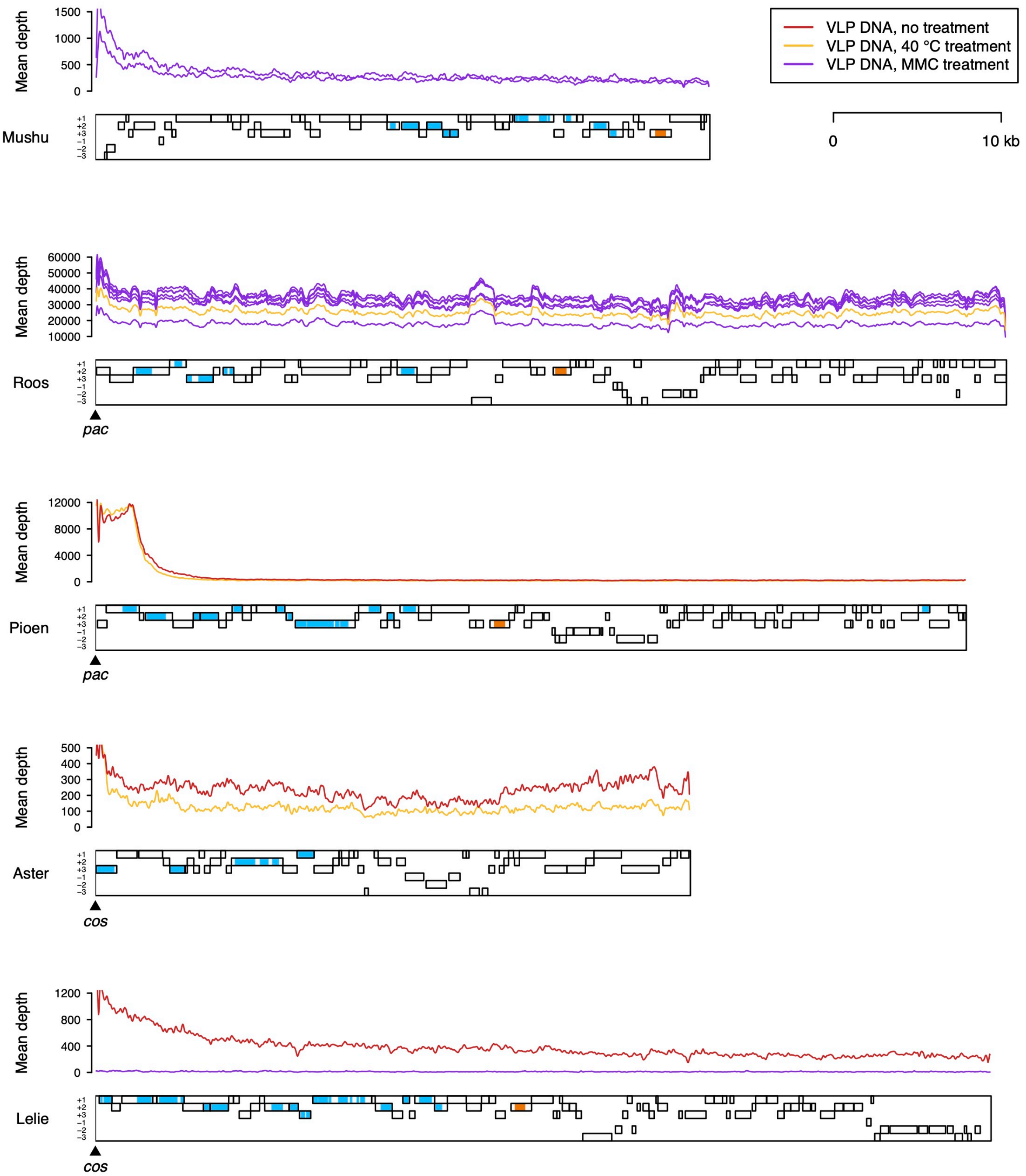

Figure S4. Coverage of phage genomes by virus-like particle reads. Genome maps of the five active phages are shown (see Figure 4 legend for details) with the positions of the predicted *pac* and *cos* sites indicated. Colored lines above the genome maps depict the depth of genome coverage by VLP reads obtained after different prophage induction treatments. Depth of coverage was recorded using a 101 nt window sliding with a 20 nt step.

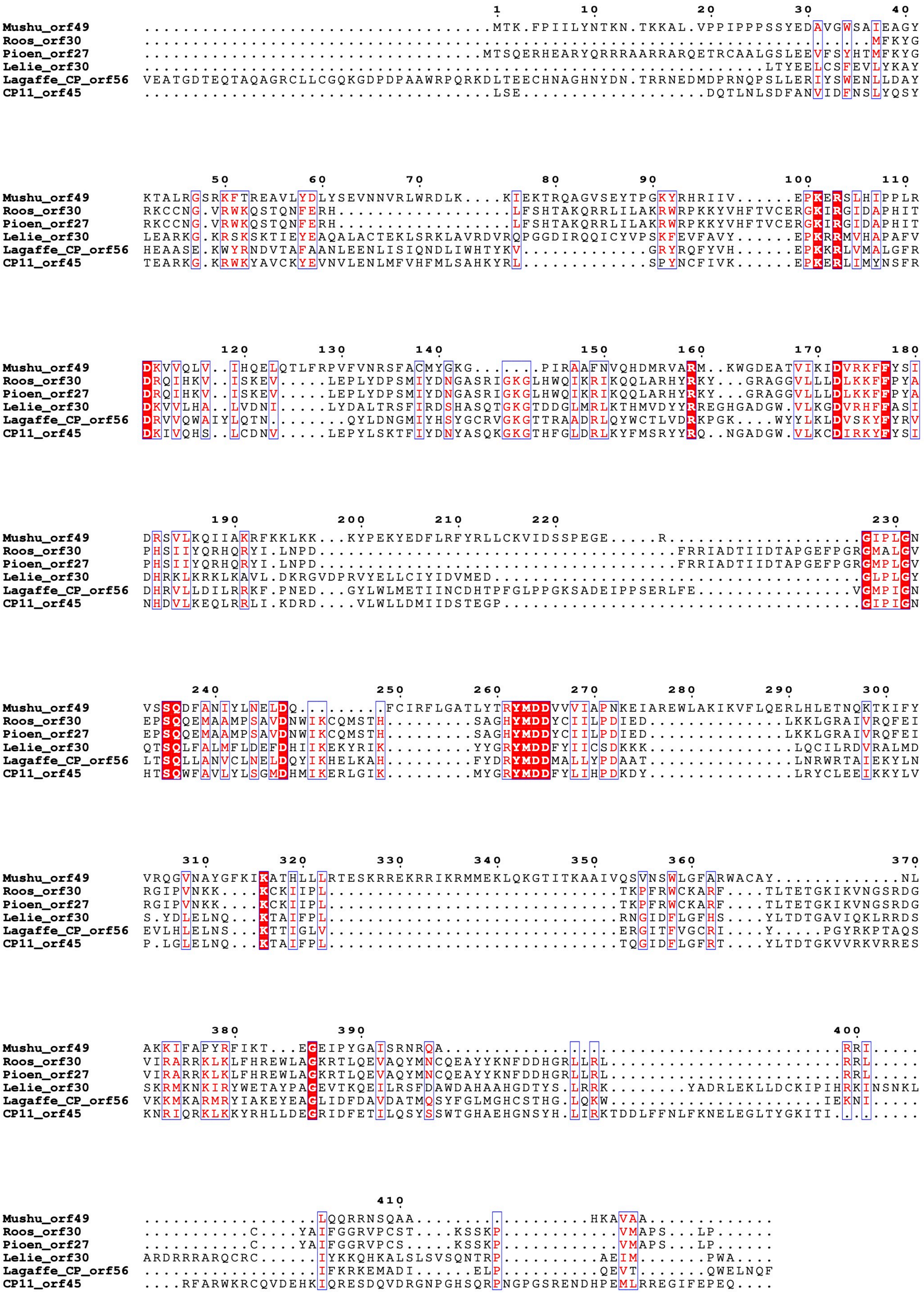

Figure S5. Alignment of reverse transcriptases encoded by phages and candidate prophages. Absolutely conserved residues are shown on a red background. Partially conserved residues are indicated in red font.

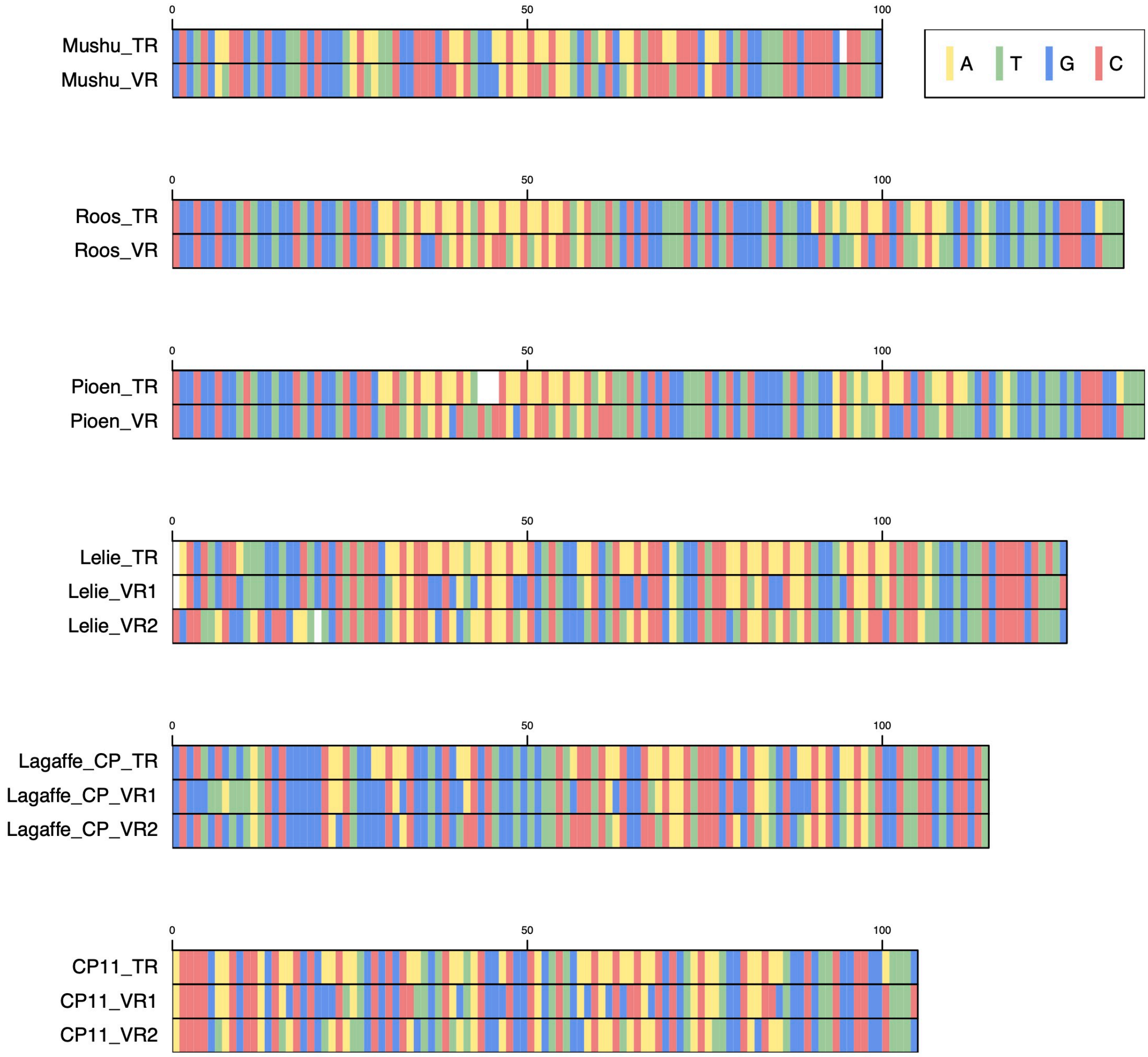

Figure S6. DGR repeats of phages and candidate prophages. Nucleotides in alignments of DGR repeats are represented by colored boxes. Empty boxes indicate gaps.

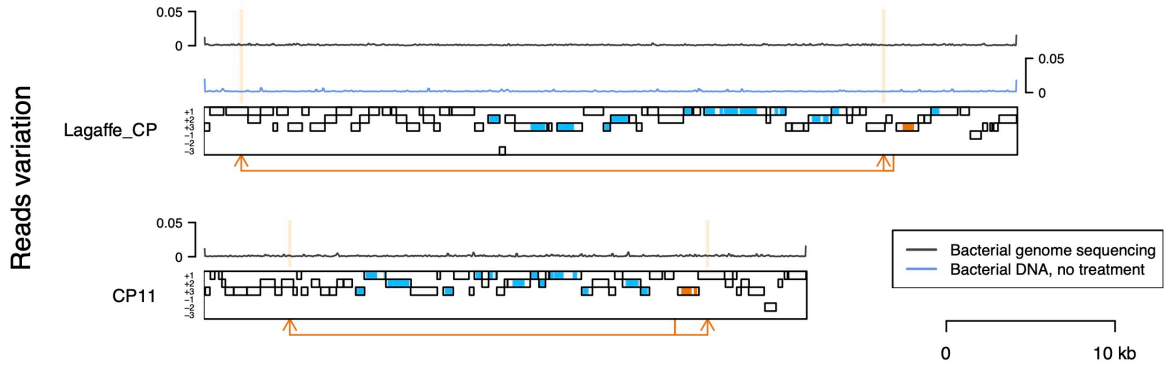

Figure S7. Nucleotide variation in sequencing reads mapped to candidate prophage genomes. Data for the two DGR-containing candidate prophages are shown (see Figure 6 legend for details).
