# Supplementary figures and images for: Identification and characterization of Faecalibacterium prophages rich in diversity-generating retroelements
Source: Microbiol Spectr. 2024 Dec 31;13(2):e01066-24. doi: 10.1128/spectrum.01066-24 (PMC11792537; doi:10.1128/spectrum.01066-24)

CP1

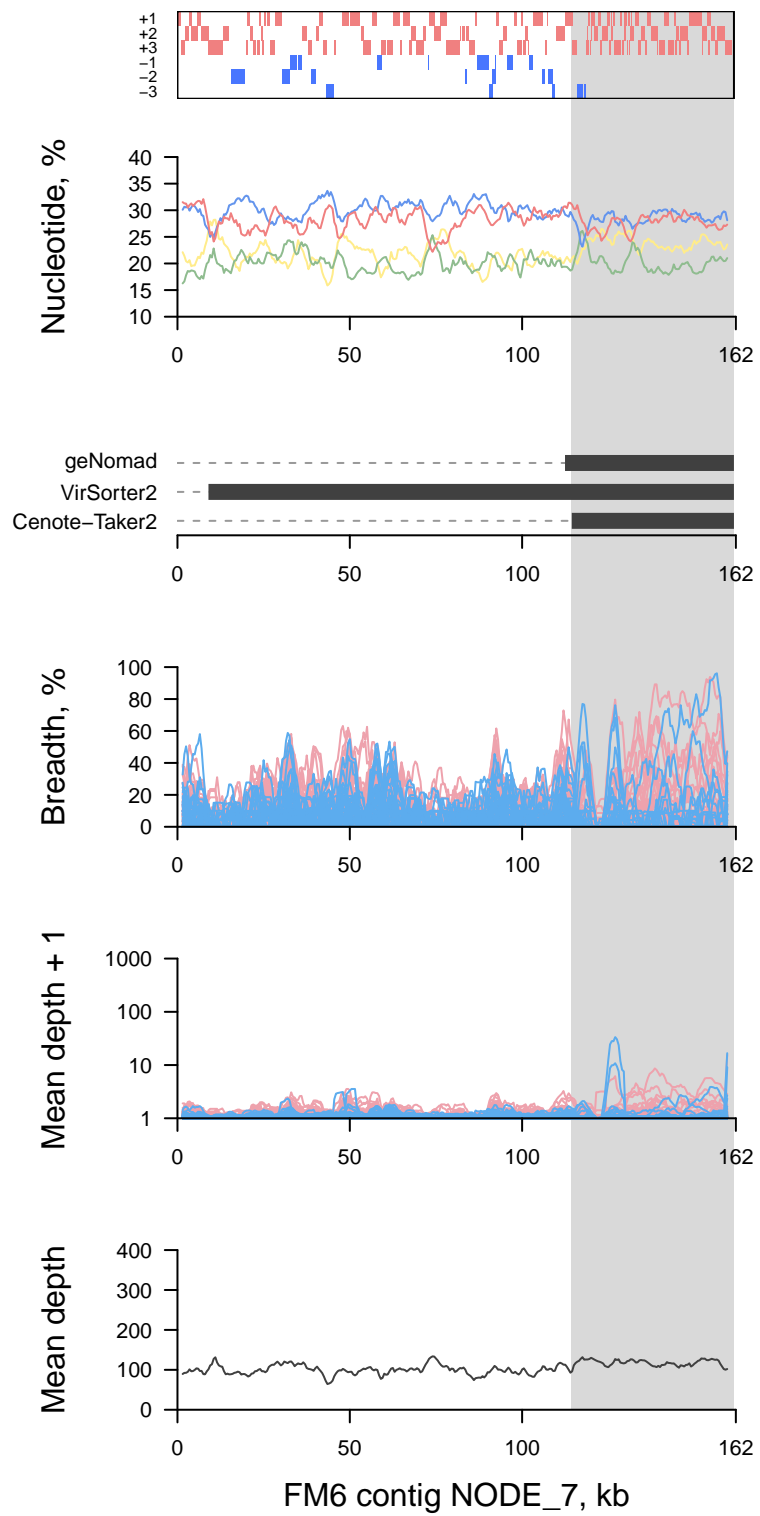

# CP2

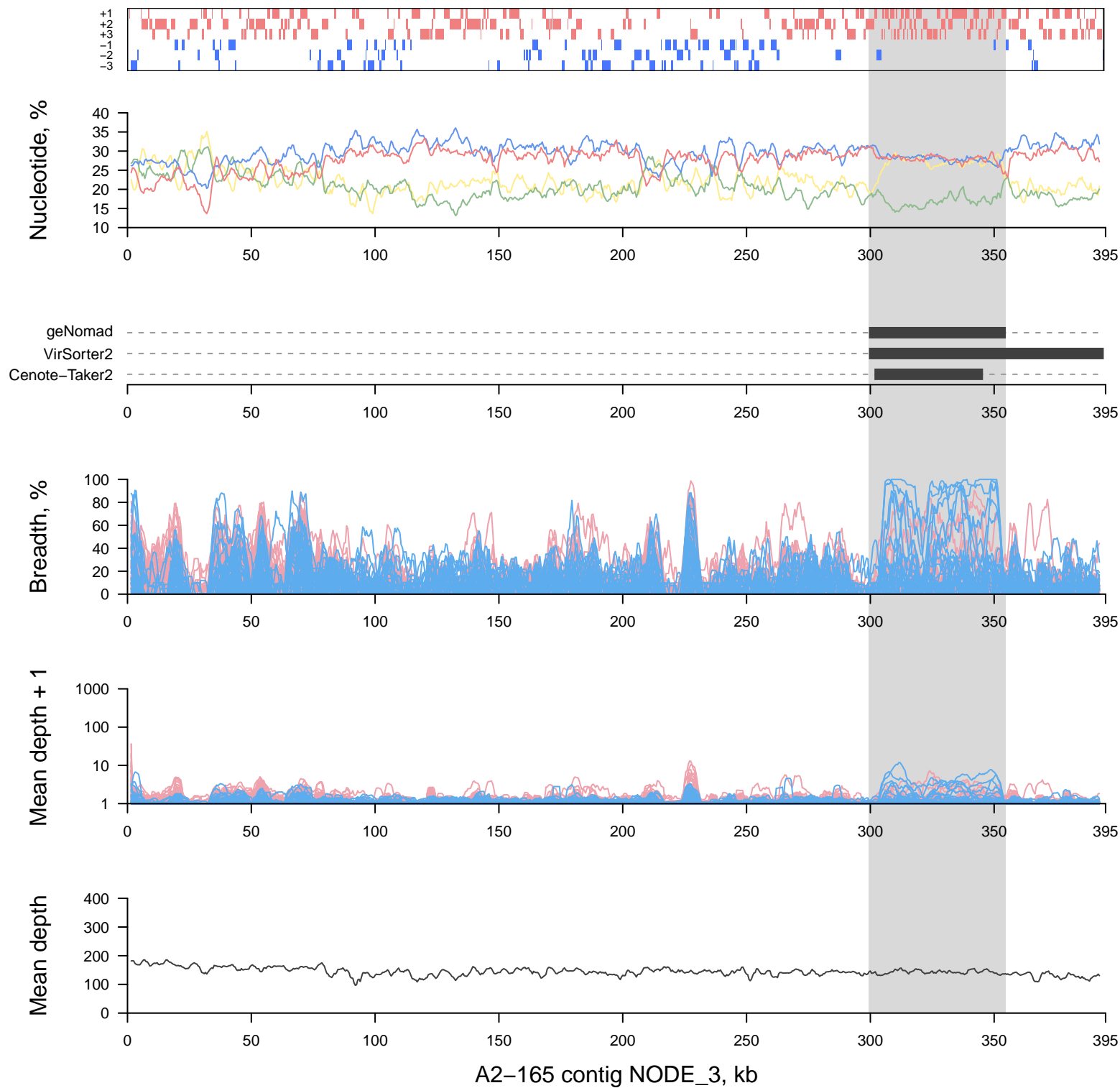

# CP3

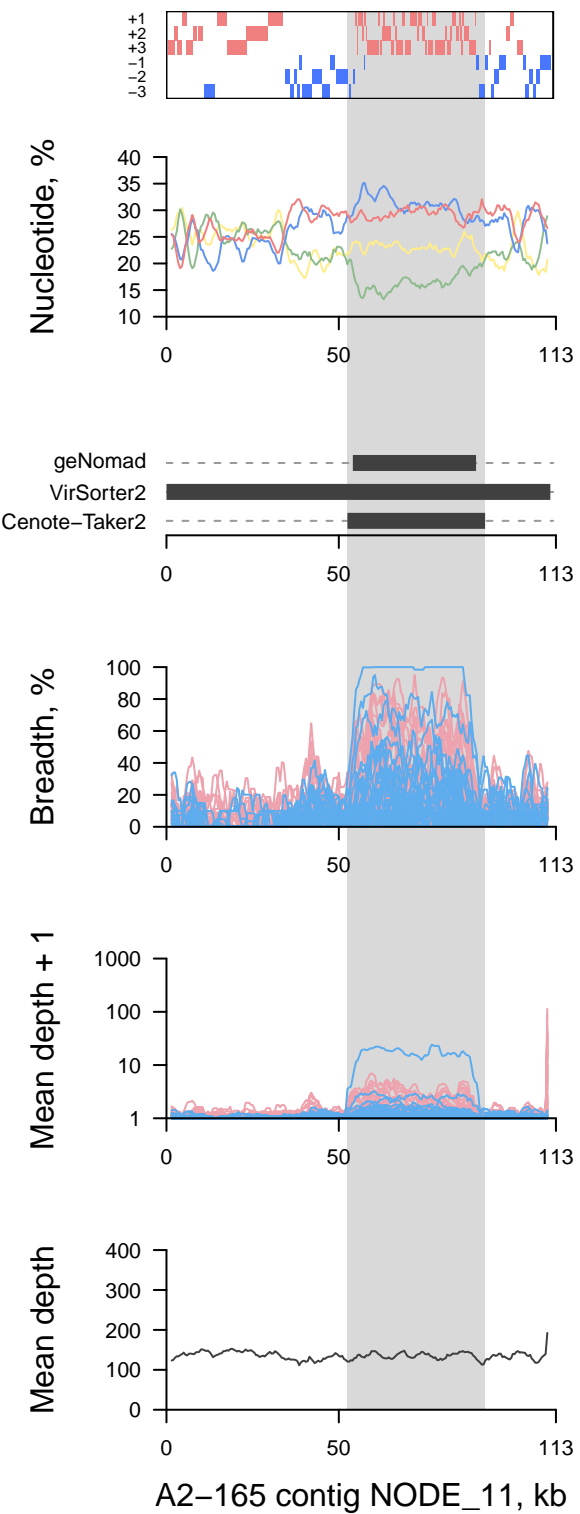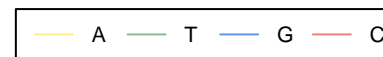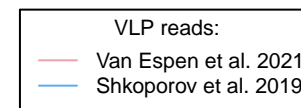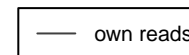

# CP4

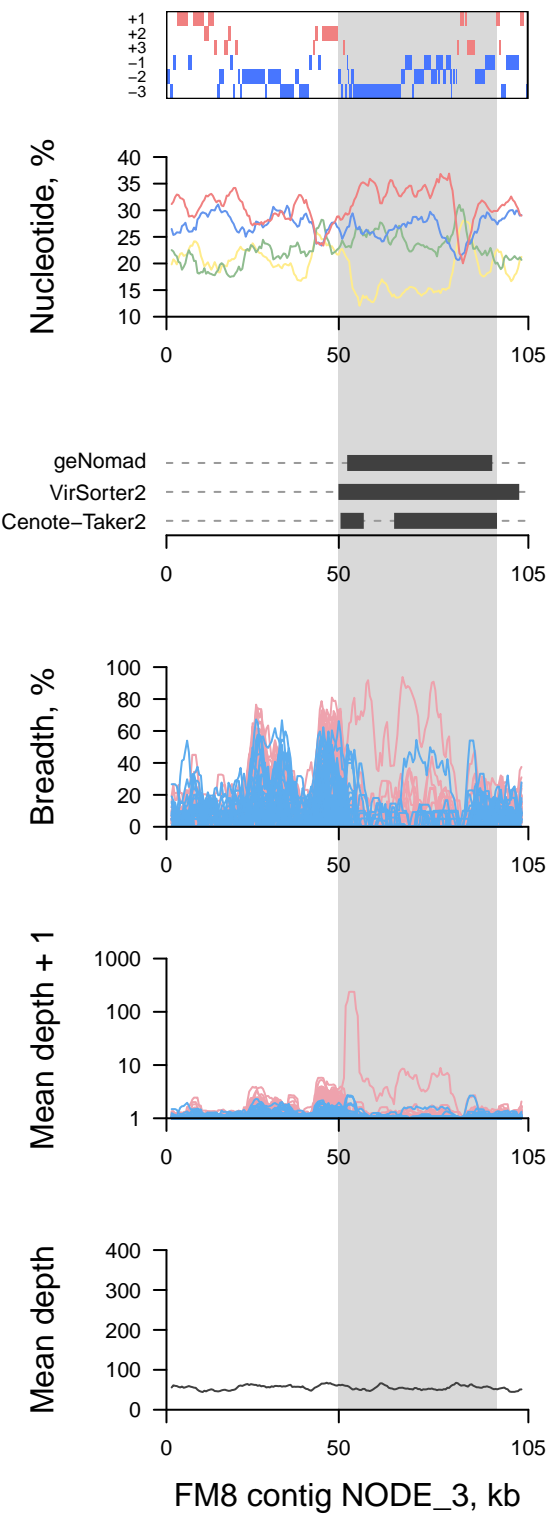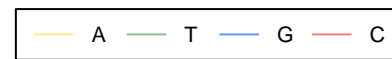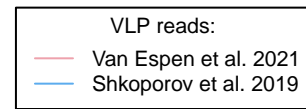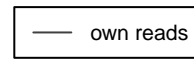

# CP5

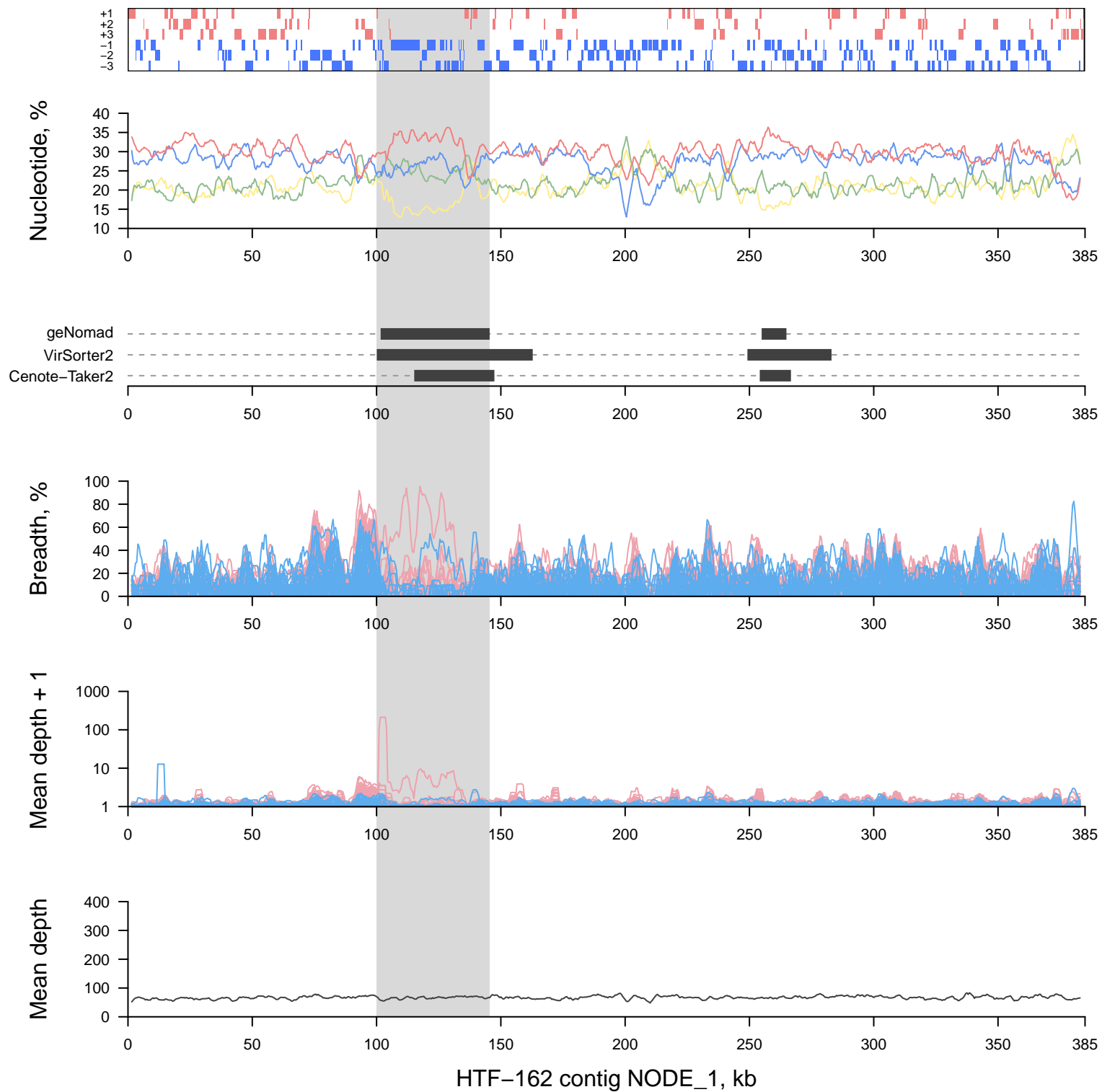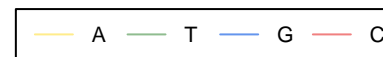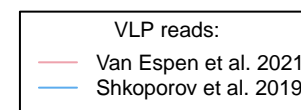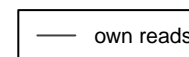

# CP6

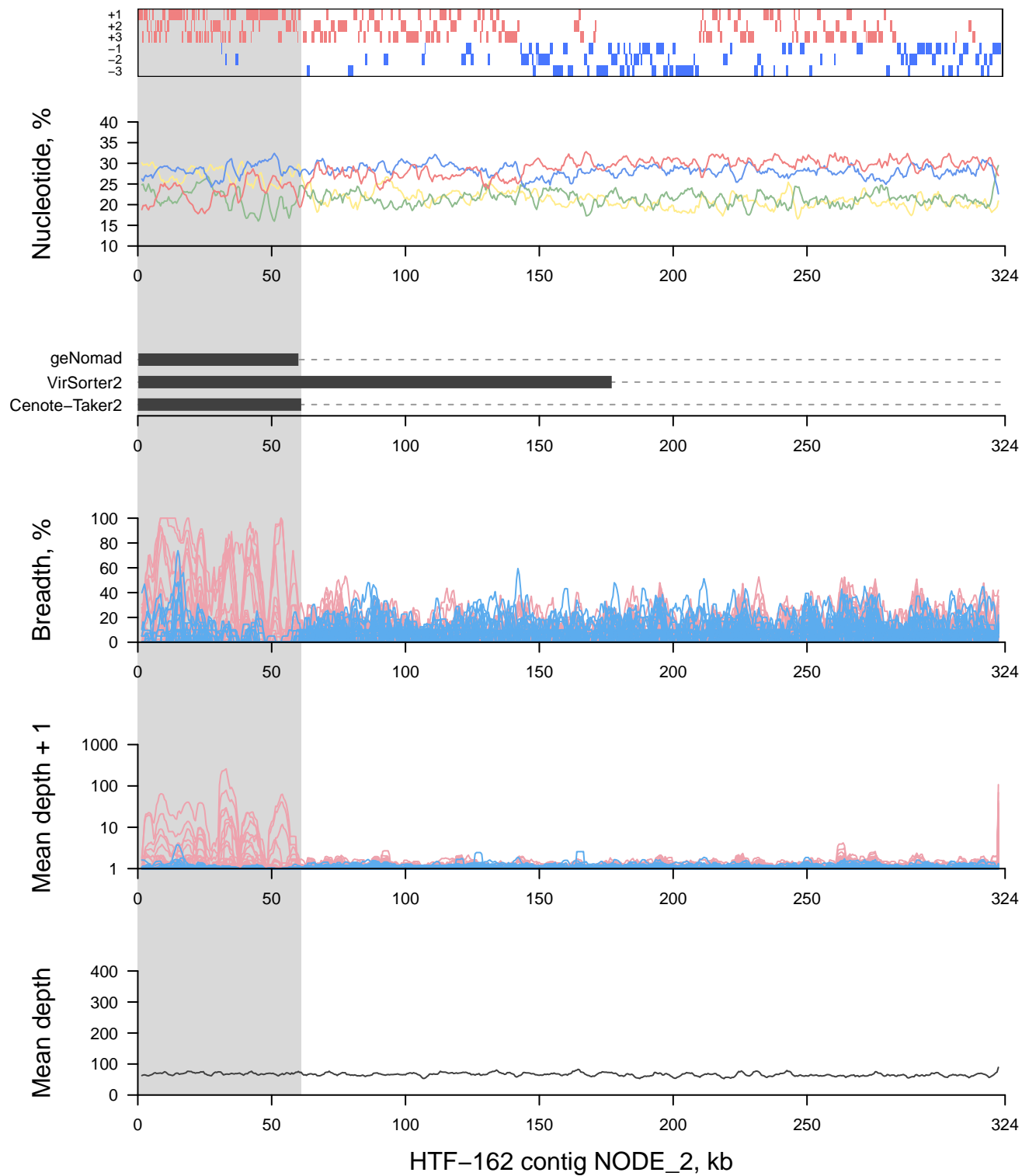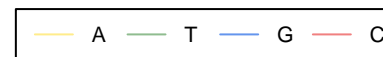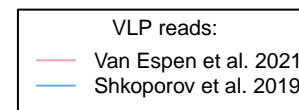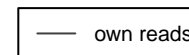

# CP7

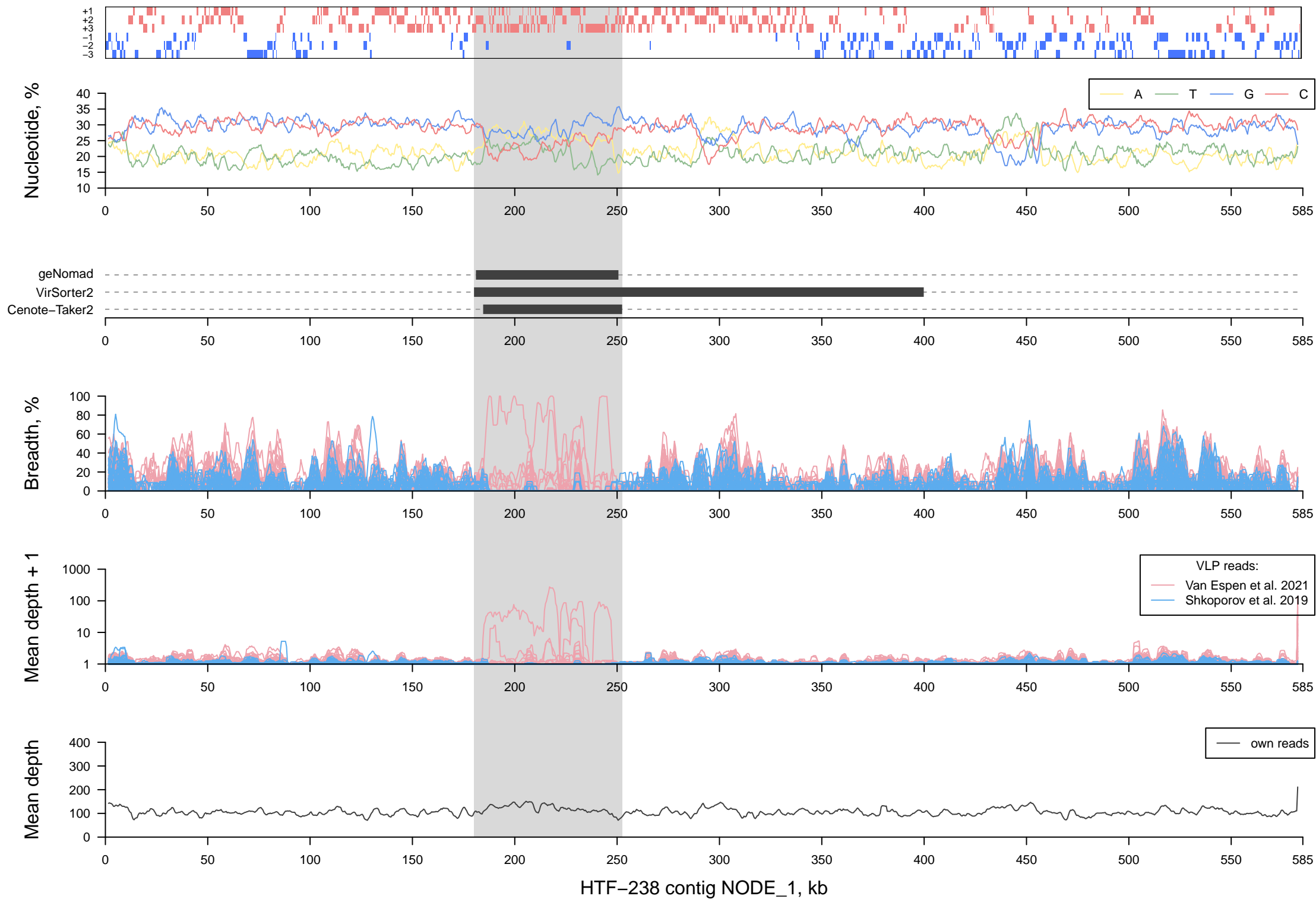

CP8

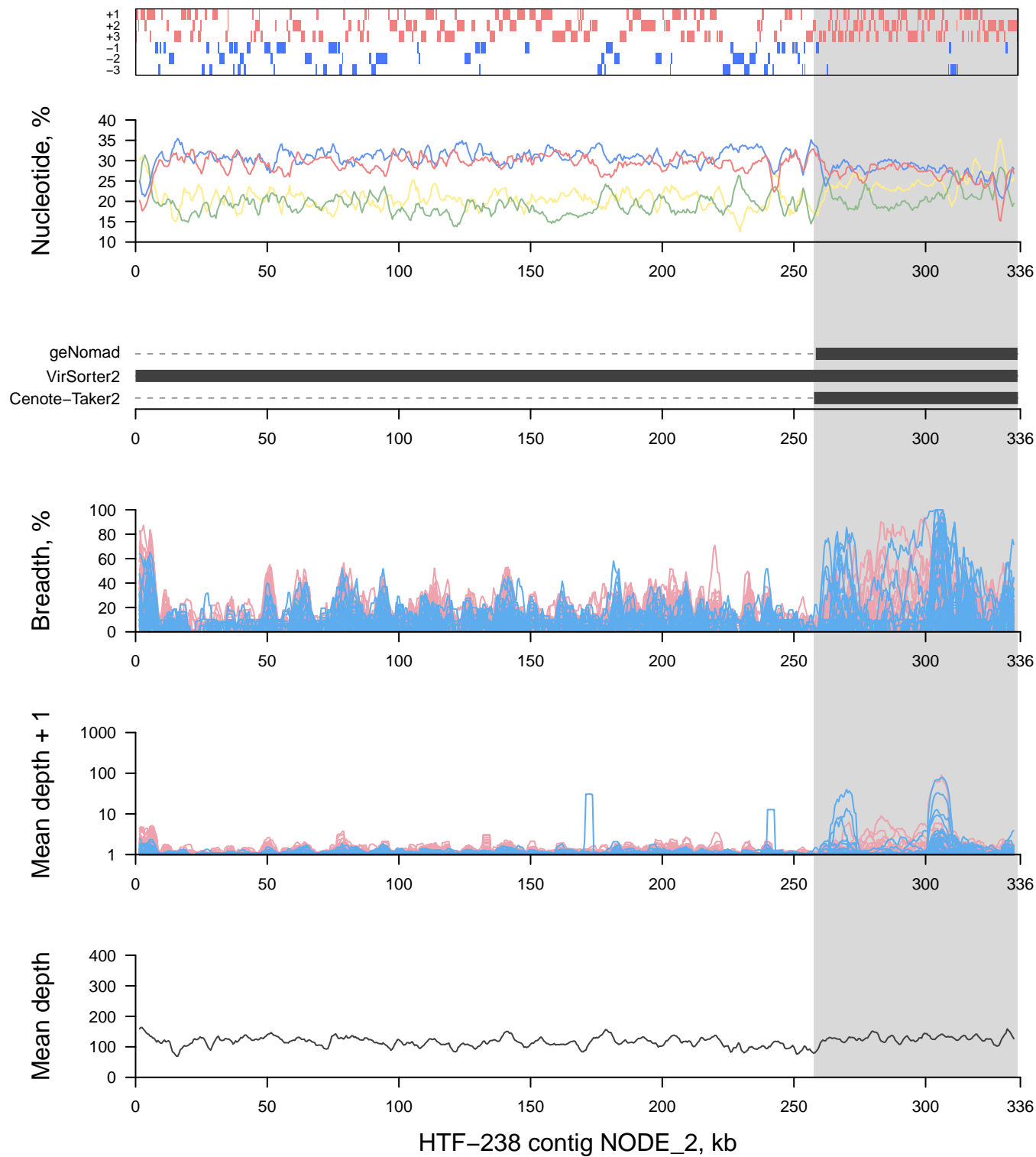

CP9

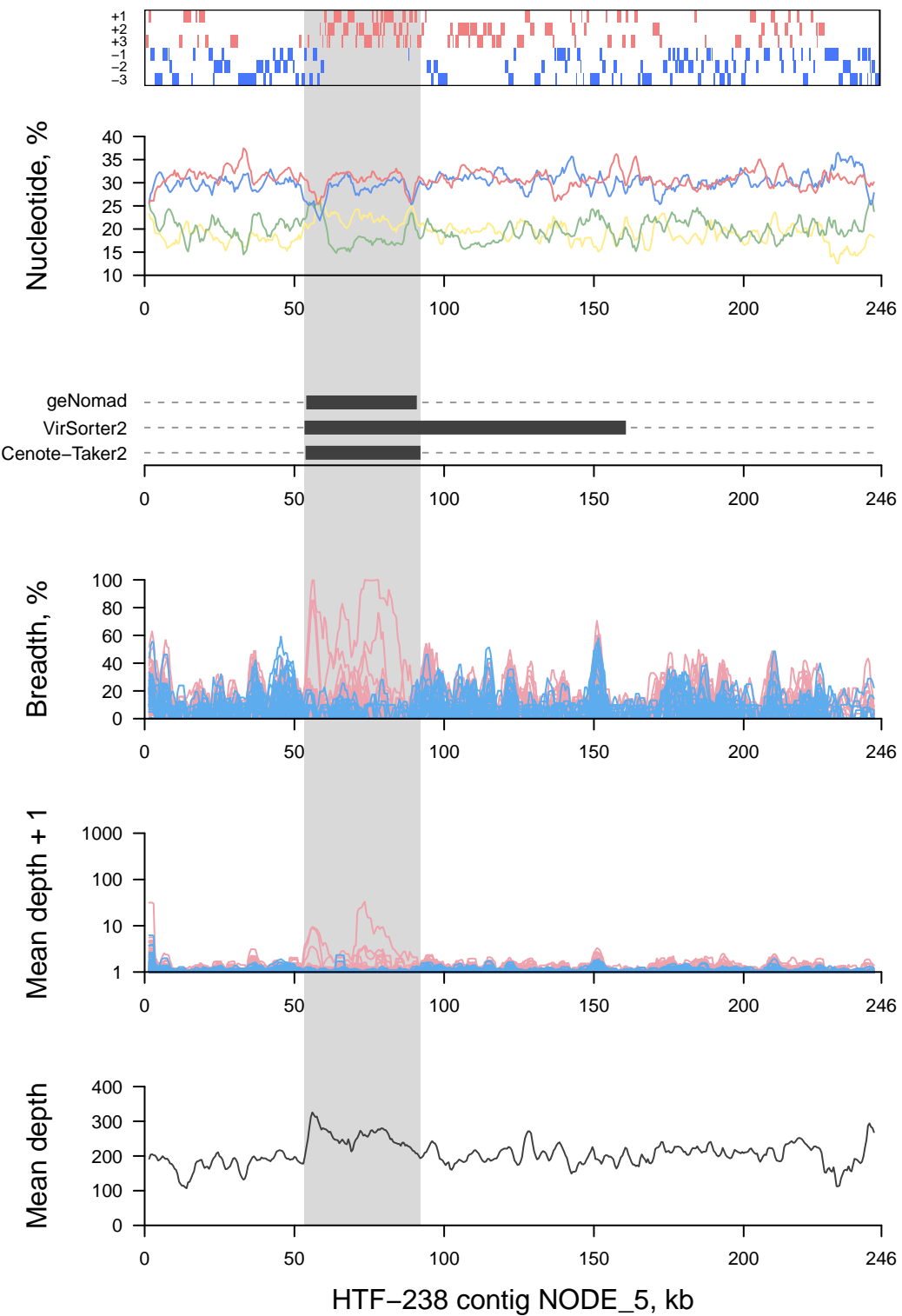

# CP10

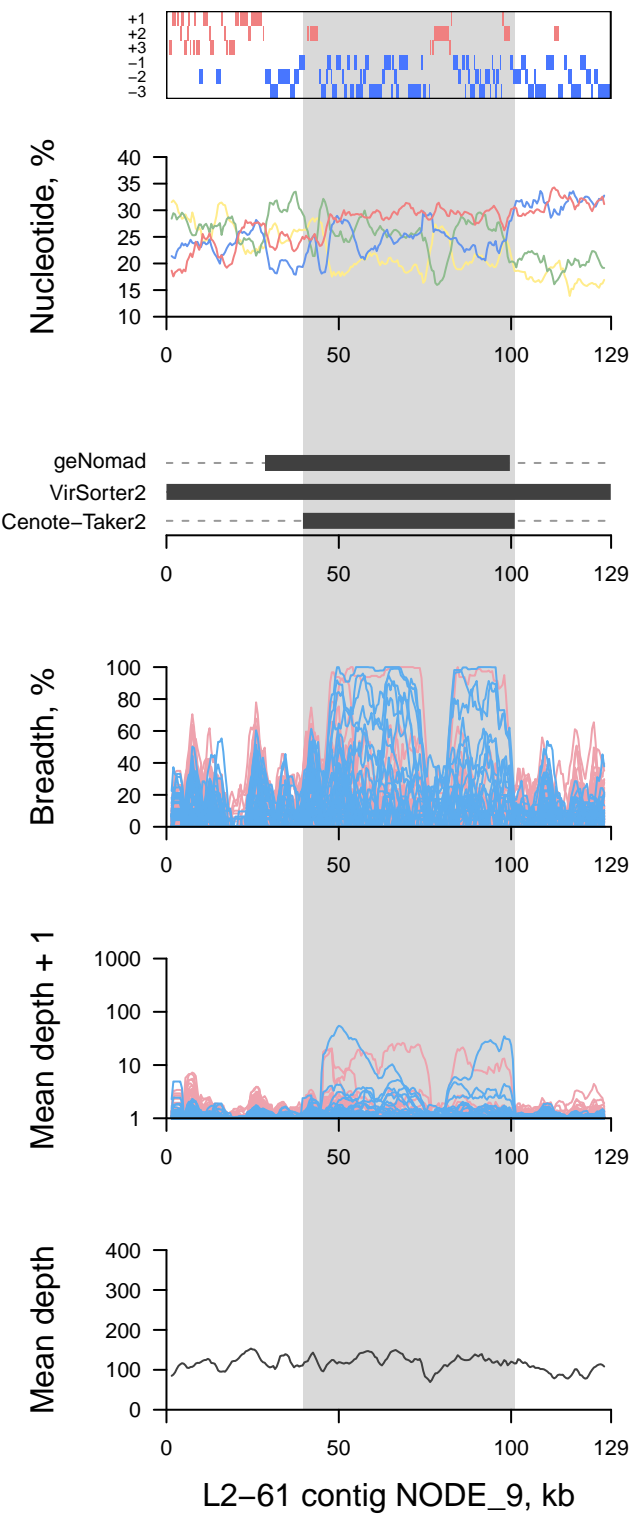

# CP11

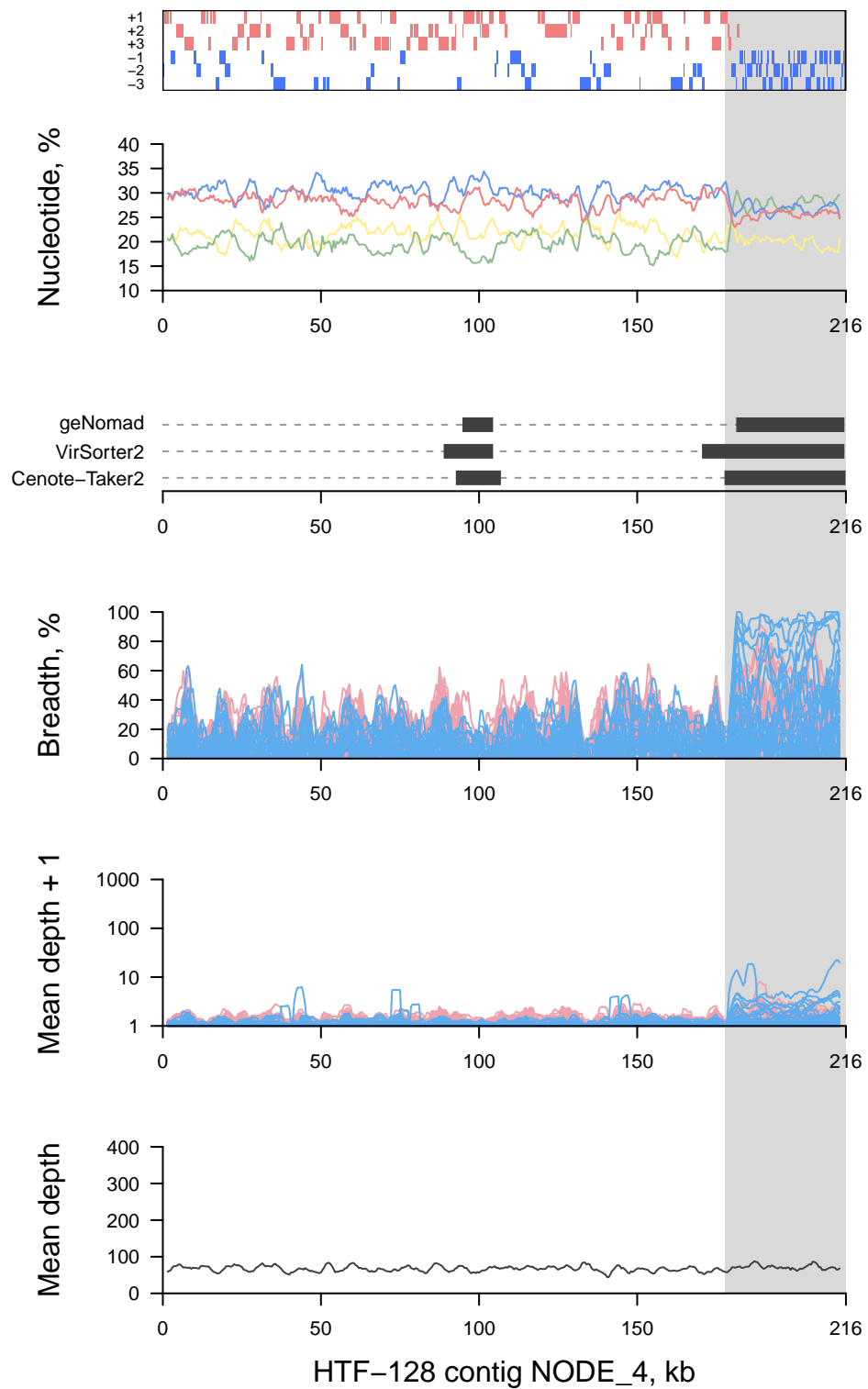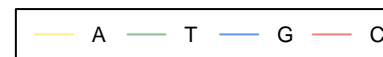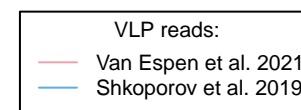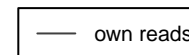

# CP12

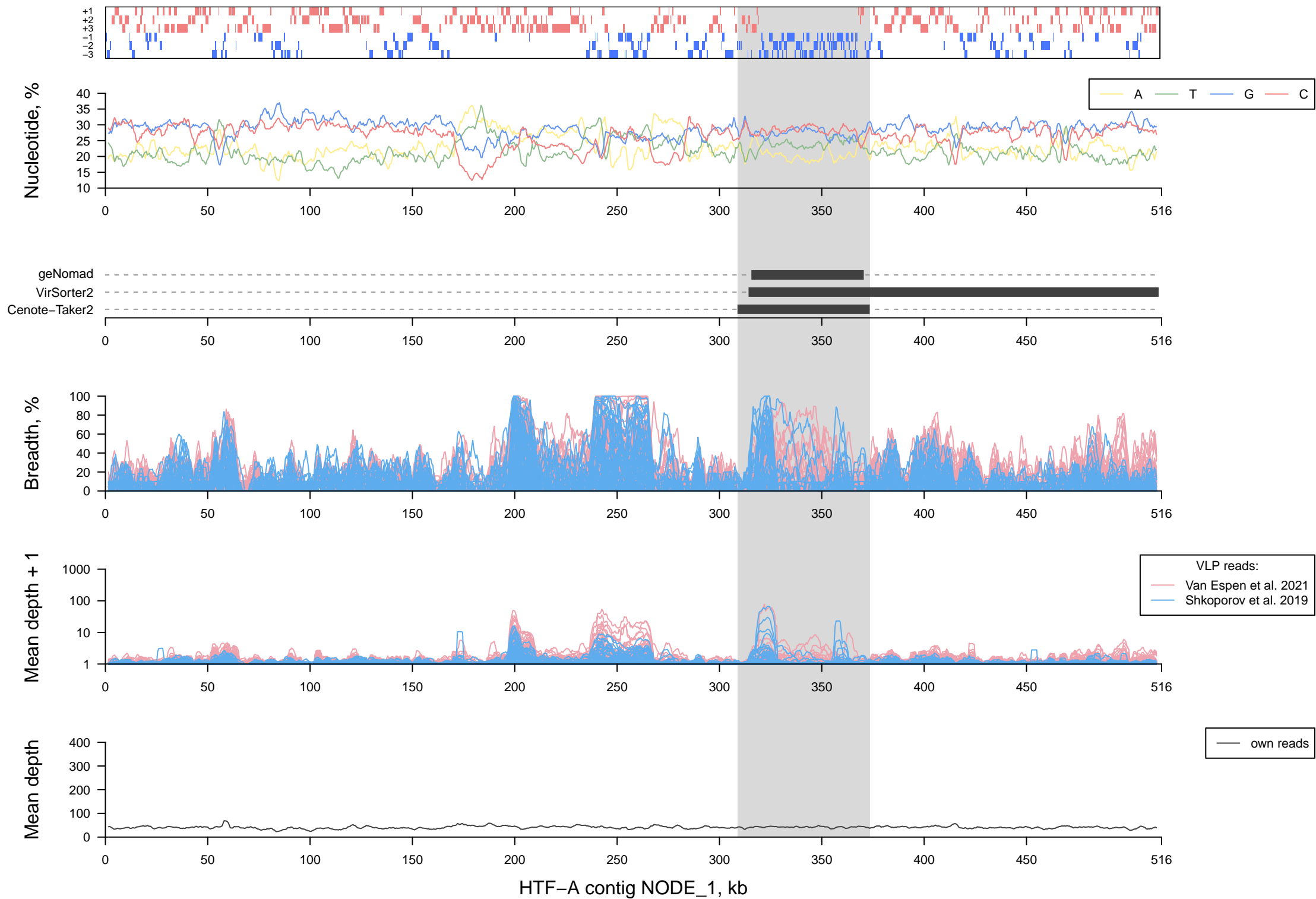

# CP13

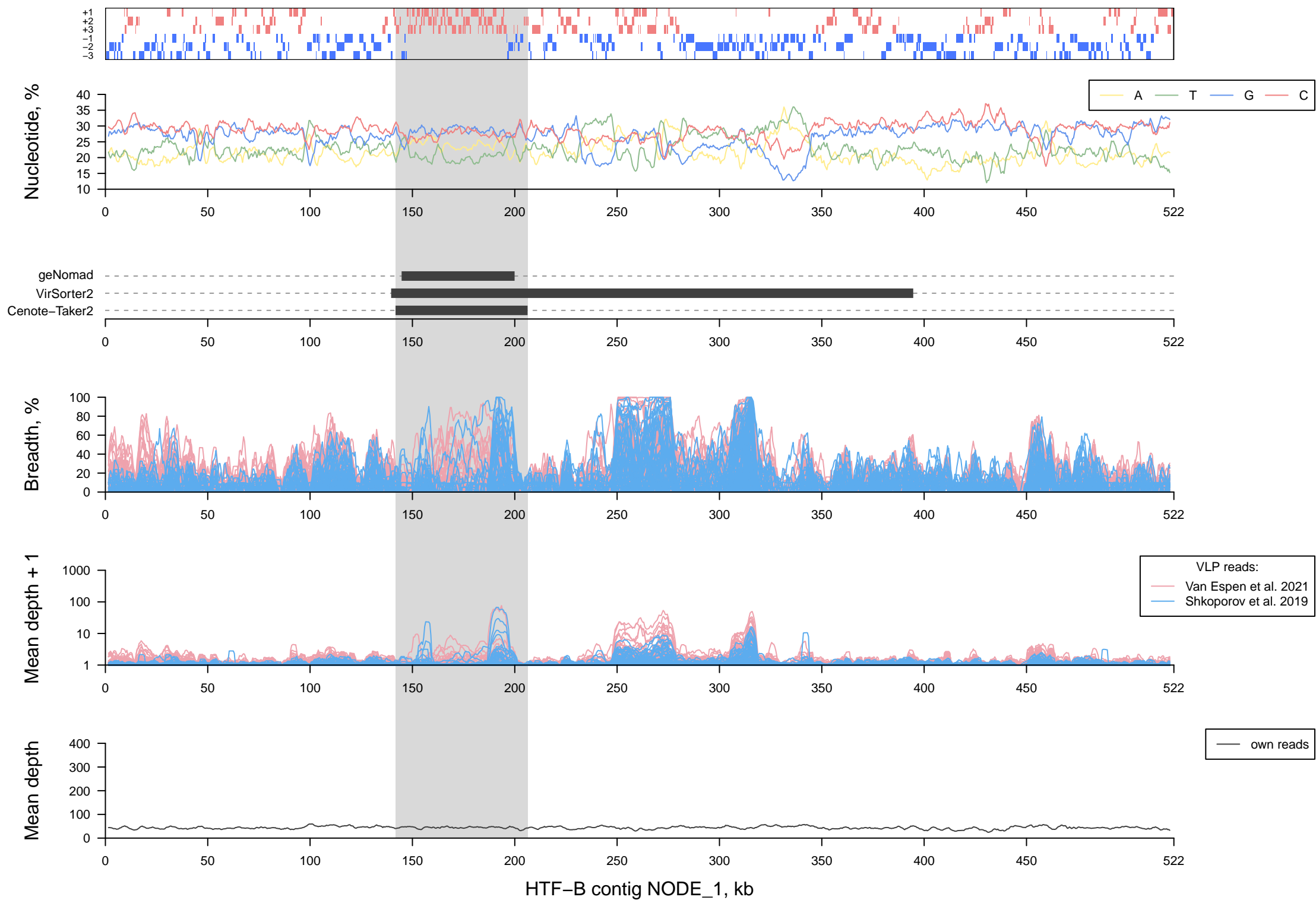

# CP14

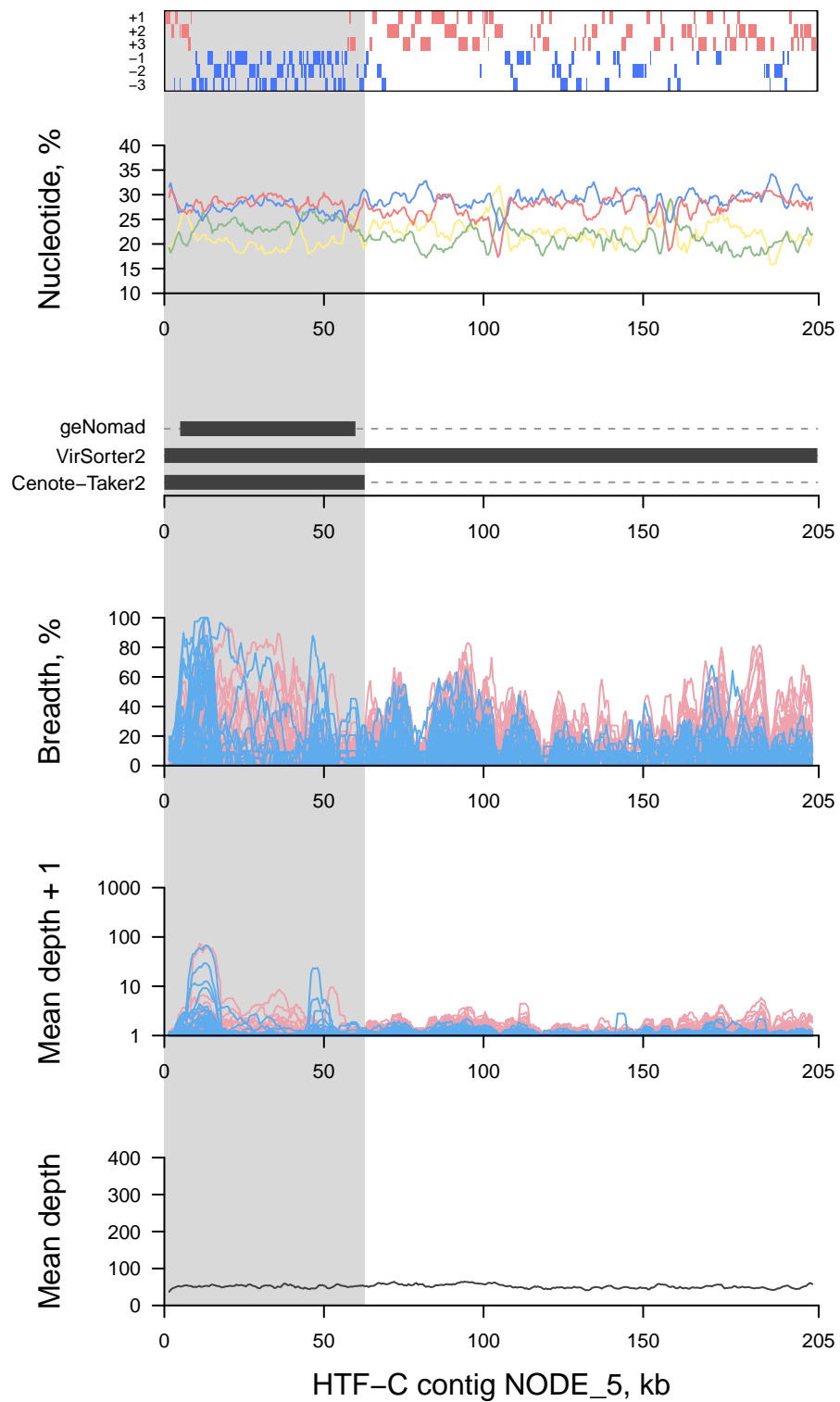

# CP15

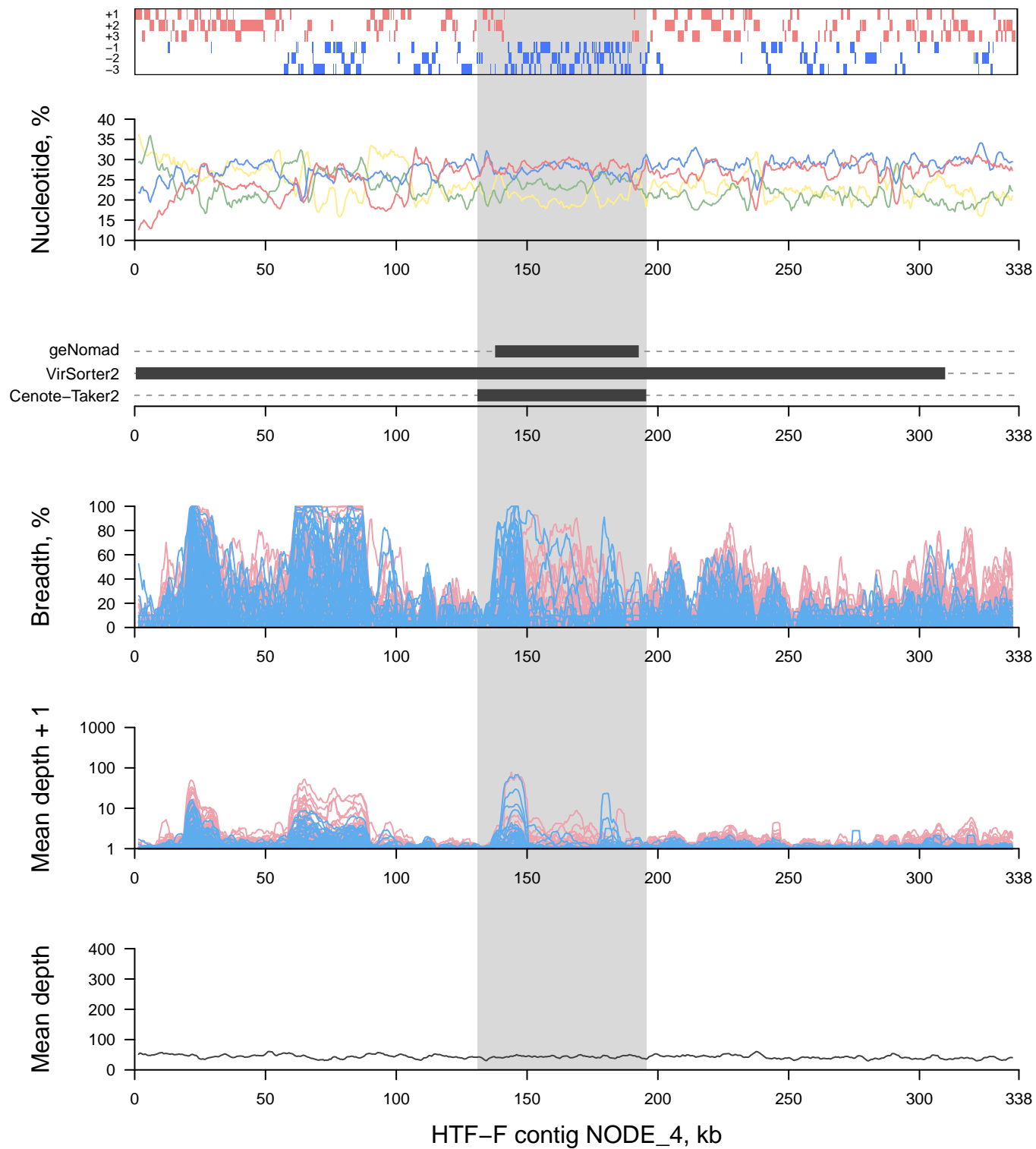

Supplement: Supplemental file S2 — Analysis of the bacterial contigs containing candidate prophages. [file spectrum.01066-24-s0003.pdf]
